# A high-resolution *N-*glycoproteome landscape of aging mouse ovary

**DOI:** 10.1016/j.redox.2025.103584

**Published:** 2025-03-07

**Authors:** Yongqi Wu, Zhida Zhang, Yongchao Xu, Yingjie Zhang, Lin Chen, Yiwen Zhang, Ke Hou, Muyao Yang, Zhehui Jin, Yinli Cai, Jiayu Zhao, Shisheng Sun

**Affiliations:** Laboratory for Disease Glycoproteomics, College of Life Sciences, Northwest University, Xi'an, 710069, PR China

**Keywords:** Glycoproteomic, Ovarian aging, Mass spectrometry, Glycosylation, LacdiNAc

## Abstract

Ovarian aging typically precedes the decline of other organ systems, yet its molecular mechanisms remain poorly understood. Glycosylation as one of the most important protein modifications has been especially unexplored in this context. Here, we present the first high-resolution glycoproteomic landscape of aging mouse ovaries, uncovering site-specific *N*-glycan signatures across subcellular components such as high proportions of complex glycans, core fucosylation, and LacdiNAc branches at the zone pellucida. We report three major glycosylation alterations in aged ovaries: the frequently changed core-fucosylation associated with cell adhesion and immune responses, the decreased LacdiNAc glycans on zona pellucida (ZP) responsible for fertility decline, and the increased sialylated glycans modified by Neu5Ac and Neu5Gc playing different roles in immune activation and responses. Integrated multi-omic analyses further highlight the unique role of glycosylation, distinct from phosphorylation, in regulating key signaling pathways, antigen processing and presentation, complement coagulation cascades, ROS biosynthetic and metabolic processes, as well as cell death. This study offers a novel glycobiological perspective on ovarian aging, broadening our understanding of its molecular mechanisms beyond traditional multi-omic approaches.

## 1 Introduction

Ovary is a vital reproductive organ responsible for the production of mature oocytes and secretion of sex hormones [1]. It also stands out as one of the first organs to display early-onset aging-associated dysfunction, characterized by functional declines including reduced ovarian reserve as well as diminished oocyte quantity and quality post the age of mid-30s [2,3]. Ovarian aging leads to estrogen deficiency, which not only directly affects the tissues and organs with estrogen receptors, such as the ovary, endometrium, vaginal epithelium, skin, hypothalamus, and urinary tract, but also influences other aspects of the organism, including the cardiovascular, musculoskeletal, immune systems, emotional and sleep patterns, cognitive ability, and energy metabolism [4]. Although multiple omic studies such as transcriptomics and proteomics have given us a better understanding of ovarian aging [[5], [6], [7]], it is still far from fully elucidating the molecular mechanisms of this complex process.

Glycosylation is known as one of the most common modifications of proteins in eukaryotes, being involved in the regulation of numerous life processes by affecting the structures and biological functions of glycosylated proteins [8]. Glycosylation not only plays an integral role in oocyte development, maturation, and female reproduction [9], but also facilitates the sperm-egg recognition and fertilization [10]. A large number of distinct proteins in the female reproductive system are glycoproteins. These include several hormones and growth factors essential for oogenesis and folliculogenesis, such as the follicle-stimulating hormone (FSH), luteinizing hormone (LH), and their cognate receptors secreted by the pituitary gland, the paracrine factors include the growth differentiation factor 9 (GDF9) and bone morphogenetic protein 15 (BMP15) secreted by oocytes, as well as the anti-Mullerian hormone (AMH) secreted by granulosa cells [[11], [12], [13]]. Systematically studying the states of these functional glycoproteins in normal ovary as well as their glycosylation changes with aging are essential for comprehensively illuminating the molecular landscape of ovarian aging.

The great macro- and micro-heterogeneities of glycosylation and huge diversities of glycan structures poses great challenges in comprehensive analyses of glycoproteome at both structural and site-specific levels. During the last few decades, glycomic and glycoproteomic approaches have been widely used for obtaining the information of released glycans and deglycosylated glycosite-containing peptides, respectively [14,15]. In recent years, a number of methods and software have also been developed to directly analyze intact glycopeptides (IGPs) [[16], [17], [18], [19], [20], [21], [22]], which can provide the composition information of glycans that are still attached at the glycosylation sites. In 2021, we also developed a modular strategy to determine the detailed *N*-glycan structures on each glycosite through the combined utilization of characteristic B and Y ions in tandem mass spectra (MS/MS) with low HCD collision energy [23]. The StrucGP software based on this strategy enables the de novo structural interpretation of site-specific *N*-glycans with high-throughput [23], and therefore achieved high-resolution glycoproteomic analyses of various biomedical samples.

In this study, we employed the StrucGP-based *N*-glycoproteomic approaches to generate the first high-resolution glycoproteome map of mouse ovary, and systematically investigated site-specific *N*-glycosylation changes during ovarian aging through tandem mass tag (TMT)-based quantification [24]. By analyzing glycan structures and their corresponding glycoproteins across various cellular components, biological processes and molecular functions, we uncovered distinct aberrations in site-specific *N*-glycans in aged ovaries. Through analyzing *N*-glycan structures that changed substantially with aging, we further identified three major age-related *N*-glycan features as well as many characteristic *N*-glycan changes that were associated with different pathways or biological processes. Furthermore, we integrated proteomic data to clarify whether these glycopeptide changes occurred at the protein expression level or site-specific glycosylation level. By integrating with phosphoproteomic data, we also identified signaling pathways associated with *N*-glycosylation and shown that glycosylation might play distinct roles other than phosphorylation in signaling pathways. These findings offer novel glycobiology insights into the mechanisms and potential therapeutic targets of ovarian aging.

## 2 Results

### 2.1 Multi-dimensional sub-proteomic analyses of aging mouse ovary

**Fig. 1:**
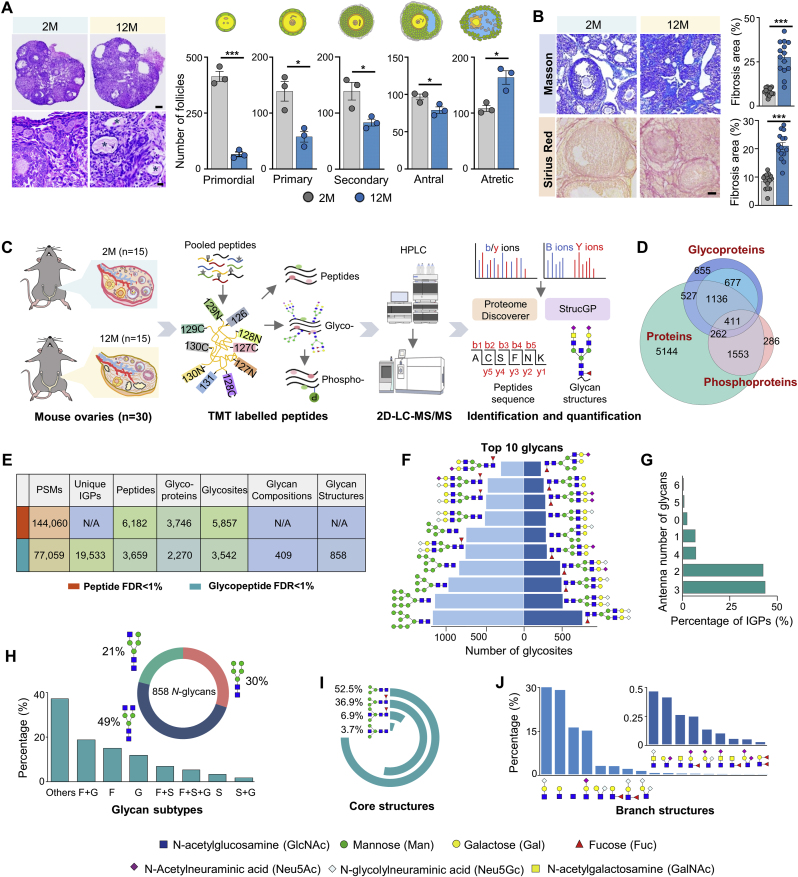
High-resolution glycoproteomic, proteomic and phosphoproteomic analyses of aging mouse ovary. (**A**) Representative hematoxylin and eosin (H&E) staining of ovaries tissues from young (2-month-old) and middle-aged (12-month-old) mice. Asterisks indicate atretic follicles (left). Density quantification of different stages of follicles indicated by the morphology (right). Follicle counts were performed in triplicate. Scale bars, 200 μm (upper left); 20 μm (lower left). (**B**) Masson's trichrome (upper) and Sirius red staining (lower) of ovarian tissues from 2- and 12-month-old mice, illustrating structural changes with aging. Scale bar, 50 μm (**C** to **F**) Quantitative glycoproteomic, proteomic and phosphoproteomic analyses on two age groups of mouse ovaries, including (C**)** the workflow, (D) numbers and overlap of identified proteins across three sub-proteomic data, (E) details of glycoproteomic results, and (F**)** ten most prevalent glycans in mouse ovaries ranked by the numbers of modified glycosites including (left panel) or excluding oligo-mannose glycans (right panel). Identification of intact glycopeptides and glycoproteins were controlled by FDR <1 % at the glycosite-containing peptide level (the outer blue circle, D; upper, E) or at both glycosite-containing peptide and glycan levels (the inner blue circle, D; lower, E). (**G** to **J**) Distribution of different modules of site-specific *N*-glycans identified from mouse ovaries, including branch numbers (G), glycan types (H), fucosylated and sialylated glycans (**H**), core structures (**I**), and branch structures (**J**). The percentages were calculated based on unique glycopeptides containing each type of glycan structural feature vesus total glycopeptides. Data presented as mean ± s.e.m. ∗*P* < 0.05, ∗∗*P* < 0.01, ∗∗∗*P* < 0.005 by two-tailed *t*-test (A, B). TMT: tandem mass tag. FDR: false discovery rate. PSMs: peptide-spectrm matches. IGPs: intact glycopeptides. F: fucose; G: Neu5Gc; S: Neu5Ac.

We collected ovary tissues from young (2-month-old, n = 15) and middle-aged mice (12-month-old, n = 15), which represented around 20- and 45-year-old of human females, respectively [25]. Since the reproductive senescence transition of mice usually occurs around 9–12 months of age [26], the middle-aged mice used here should be at the stage of reproductively senescent with a sharp decline in fertility, which are suitable for ovarian aging studies. Experimentally, although the ovarian weight increased with body weight in 12-month-old mice compared to 2-month-old mice, their ovarian index was even slightly reduced (Fig. S1A). Histologically, the numbers of follicles were decreased at all different developmental stages of aged ovaries compared to the young group, accompanied by an elevated number of atretic follicles (Fig. 1A; Fig. S1B). In addition, significant fibrosis was observed in the stroma of aged ovaries, as indicated by Masson's trichrome (MT) and Sirius Red (SR) staining (Fig. 1B). These data confirmed the occurrence of aging-related changes in the ovaries of middle-aged mice.

Fifteen ovary samples from each age group were combined into five pooled samples, followed by multi-dimensional sub-proteomic analyses, including quantitative glycoproteome, phosphoproteome, and global proteome. Proteins extracted from each pooled sample were digested into peptides and labeled with one channel of 10-plex TMT reagents. After combining ten TMT-labeled samples, a small portion of pooled sample was directly used for quantitative proteomic analyses, while the remaining portions underwent sequential enrichment of intact glycopeptides and phosphopeptides (Fig. 1C). Each of three samples including glyco-, phospho-, and global peptides was separated into 24 fractions prior to LC-MS/MS analyses.

Within 1 % false discovery rate (FDR), we identified 9033 proteins, 2270 *N-*glycoproteins (FDR<1 % at both the glycosite-containing peptide and glycan levels), and 2590 phosphoproteins (Fig. 1D; Table S1). Notably, when FDR was controlled only at the glycosite-containing peptide level (regardless of their attached glycans), the numbers of identified glycoproteins were increased to 3746. The majority of peptides in the sub-proteomic data ranged in length from 6 to 20 amino acids, indicating that sample preparation reached the standard (Fig. S1C). Over 86 % of glycoproteins and 83 % of glycopeptides overlapped among triplicate LC-MS analyses of a single fraction, demonstrating high reproducibility of the mass spectrometry data (Fig. S1D). By summing up three sets of omics data, a total of 10,028 proteins were identified in young and middle-aged mouse ovaries, representing an in-depth glycoproteomic and proteomic landscape of aging mouse ovary.

### 2.2 High-resolution glycoproteome landscape of mouse ovary

As mentioned above, when glycoproteome data were controlled by the FDR<1 % and at least 5 b/y ions at the glycosite-containing peptide level, we identified 6182 peptides certainly modified by glycans from 144,060 pairs of oxonium ion-containing spectra, representing 5857 *N*-glycosites from 3746 *N*-glycoproteins (Fig. 1E). After further filtering the results by FDR<1 % at the glycan level, a total of 19,533 unique *N*-glycopeptides were identified from 77,059 high-quality MS/MS spectra, comprising 858 *N*-glycans and 3542 *N*-glycosites from 2270 *N*-glycoproteins (Fig. 1E; Table S2). Although most identified glycoproteins only had one *N*-glycosite, we did identify 28 % of glycoproteins that contained 2 to 33 *N*-glycosites (Figs. S2A and S2B). Motif analysis revealed that *N*-glycosylation occurred more frequently at the N-X-T motif than the N-X-S motif (Figs. S2C and S2D), which were consistent with previous reports [26]. These glycoproteins were mainly located at the cell surface, extracellular matrix, endoplasmic reticulum, Golgi apparatus, and lysosome, etc., performing functions such as cell adhesion, signaling, immune system processes, and reproduction **(**Fig. S2E).

By classifying different *N*-glycan structures based on their compositions, we found that nearly half of the glycan compositions were further identified as 2–12 different glycan isoforms (Fig. S2F), suggesting the high-resolution interpretation of N-linked glycans at the site-specific and structural levels. Based on the numbers of modified glycosylation sites, the top ten ovarian glycans were either sialylated bi-antennary glycans (N4H5F1G2, N4H5G2, N4H5F1G1, N4H5F1S1G1, N4H5S1. N: HexNAc; H: Hex; F: Fucose; S: Neu5Ac; G: Neu5Gc) or oligo-mannose glycans (N2H5–N2H9, also known as Man5-Man9). After excluding the oligo-mannose glycans, three additional bi-antennary glycans (N4H5G1, N4H5F1S2, N4H5F1) and two hybrid glycans (N4H6F1G1, N4H6F1S1) were also highly modified on a large number of glycosites (Fig. 1F). In addition, although the majority of glycans in the mouse ovary were bi- (42 %) and tri-antennary glycans (43 %), we did observe several *N*-glycans with 5–6 antennas modified by sialic acid or antenna-fucose (Fig. 1G). Interestingly, the glycans with 5–6 antennas were declined substantially with age and were strongly associated with fertility regulation (Fig. S3).

Based on modular structures of *N*-glycans [23], these glycans were comprised of four types of core structures and seventeen branch structures with three glycan subtypes (Fig. S4). Interestingly, the majority of glycosites were occupied by complex glycans (9610 unique IGPs, 49 %), followed by oligo-mannose (5909 IGPs, 30 %) and hybrid glycans (4014 IGPs, 21 %) (Fig. 1H). These results were different from previous reports that more than half of glycosites were modified by oligo-mannose glycans in most tissue samples [27,28]. Additionally, 17 %, 15 %, and 5.2 % of the glycosites were modified by glycans with sialylation (Neu5Ac and/or Neu5Gc), fucosylation, and both, respectively (Fig. 1H). Among four core structures, the typical core structure N2H3 accounted for 52.5 % of all unique glycopeptides, followed by fucosylated core structure (36.9 %), and bisected core structure with and without core fucosylation (6.9 % and 3.7 %, respectively) (Fig. 1I). Among 17 branch structures, the LacNAc terminated with Neu5Gc (NHG) and sole LacNAc (NH) accounted for 29.8 % and 28.8 % of unique IGPs, respectively. Sole HexNAc and terminal-sialylated LacNAc with Neu5Ac (NHS) took up 16 % and 15 % of IGPs. In addition, the LacdiNAc-containing branches (N2) with/without Neu5Ac/Gc or fucose (N2S, N2G, N2F), as well as Lewis^x/a^ (NHF) were also identified at a relatively low abundance (Fig. 1J).

### 2.3 Different subcellular localizations exhibit diverse glycan structure patterns

**Fig. 2:**
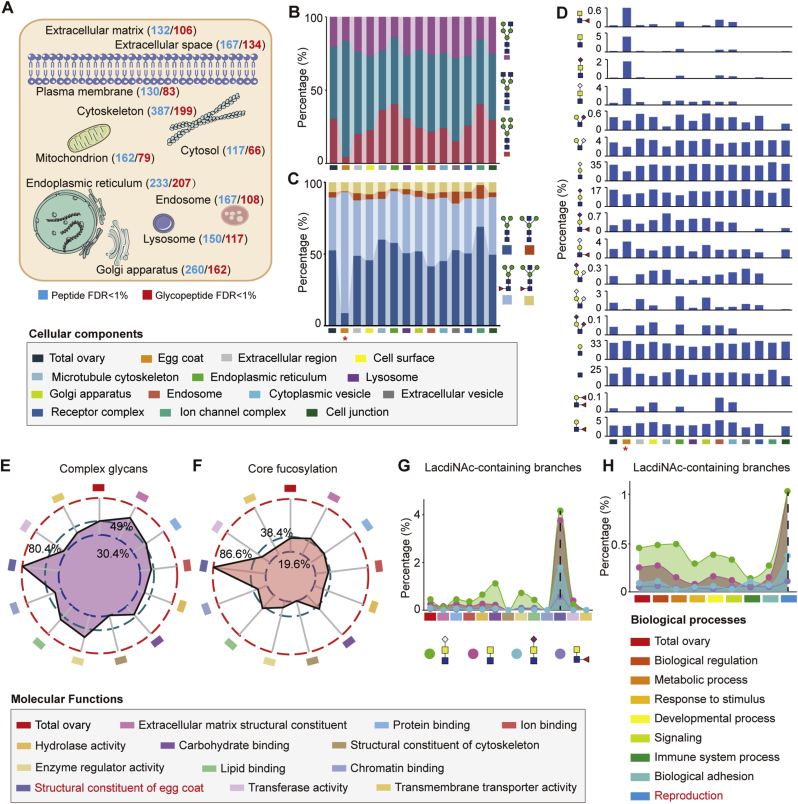
Glycan structure patterns among different subcellular localizations of glycoproteins in mouse ovary. (**A**) The numbers of glycoproteins enriched in different cellular components. The numbers of glycoproteins identified based on the FDR <1 % at the glycosite-containing peptide level and at both glycosite-containing peptide and glycan levels are highlighted by blue and red, respectively. (**B** to **D**) Comparative proportion analysis of glycan subtypes (B), core (C), and branch structures (D) across cellular components. Red asterisk symbols in panels (C) and (D) indicate entries with significantly different *N*-glycan structural characteristics compared to other terms. (**E** and **F**) Distribution of complex glycans (E) and fucosylated core structures (F) among different molecular functions. (**G** and **H**) Abundance of four LacdiNAc-containing branch types across different molecular functions (G) and biological processes (H), with enrichment observed in reproductive-related categories. Detailed quantitative data corresponding to panels E-H are provided in Fig. S5.

According to the subcellular localization from the UniProtKB database, the glycoproteins identified at the intact glycopeptide level were mainly located in the endoplasmic reticulum (207), cytoskeleton (199), Golgi apparatus (162), and lysosome (117) (Fig. 2A; Table S3). By further comparing the glycan structures among glycoproteins located at different cellular components, we found that the egg coat (also known as the zone pellucida) displayed a higher proportion of complex glycans and fucosylated core structures, complemented with much less hybrid glycans and typical core structure compared to glycoproteins located at other cellular components (Fig. 2B and C). The egg coat also exhibited a high diversity of branch structures (14/17), with a notably high abundance of LacdiNAc-containing branches but rare presence of sialylated branches (Fig. 2D). In contrast, ion channel complex had a limited number of branch structures (5/17), especially lacking branches with antenna-fucoses and/or LacdiNAc.

We also compared the structural features of *N*-glycans on glycoproteins based on different molecular functions and biological processes, and found again that *N*-glycans on glycoproteins associated with the structural constituent of egg coat were predominantly complex glycans and featured by fucosylated core structures (Fig. 2E and F), which were clearly differentiated from glycoproteins associated with other molecular functions. Additionally, much higher percentages of LacdiNAc structures were observed on glycoproteins related to the egg coat structural components and the reproductive processes compared to other related categories, which is consistent with the previous findings in cellular components, suggesting that LacdiNAc structures may be closely associated with reproductive processes (Fig. 2G and H; Fig. S5A and S5B).

### 2.4 Major site-specific glycan alterations with ovarian aging

**Fig. 3:**
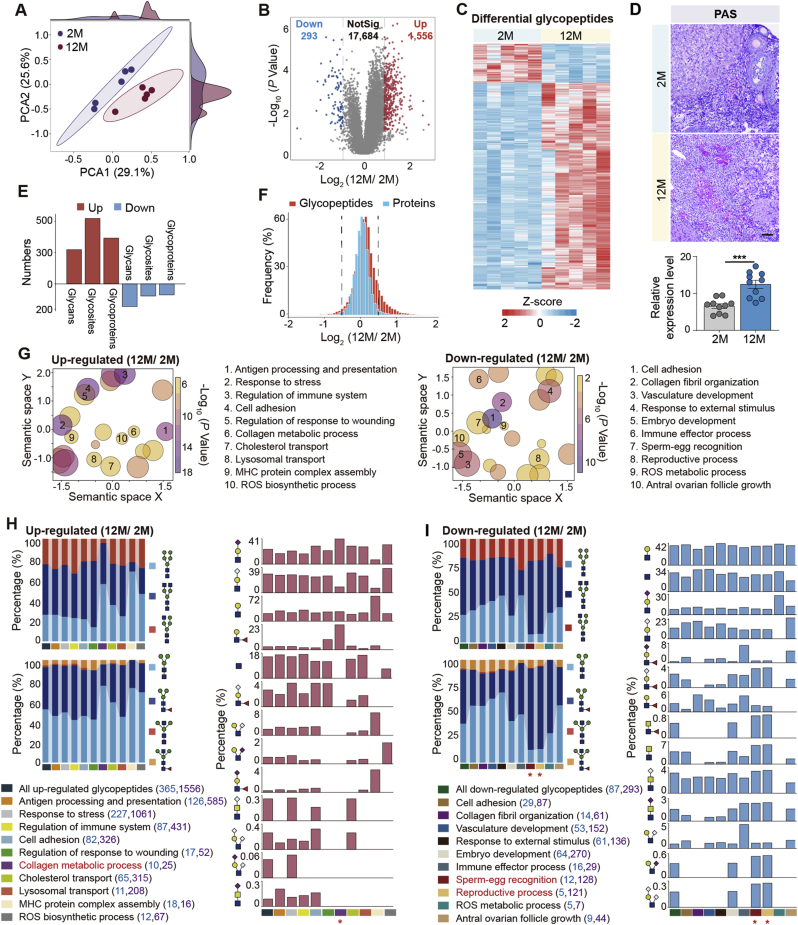

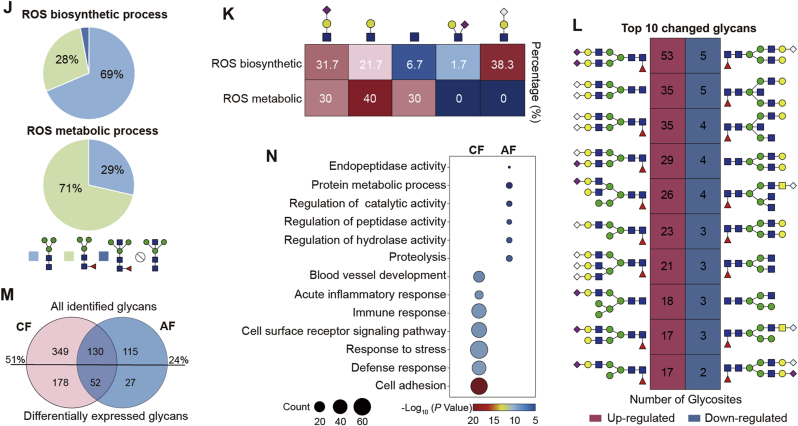
Site-specific glycosylation alterations during ovarian aging. (**A**) Principal component analysis (PCA) plot showing distinct separation between young (2-month-old) and middle-aged (12-month-old) ovarian groups. (**B**) Volcano plot highlighting significantly upregulated and downregulated glycopeptides in aged ovaries (cutoff: *p*-value<0.05 & FC > 1.5). (**C**) Heatmap showing differential expression of glycopeptides between 2-month-old and 12-month-old mouse ovaries. (**D**) Photomicrograph of Periodic Acid Schiff (PAS)-stained ovarian sections from young and middle-aged groups, visualizing glycosylation changes at the histological level. (**E**) Comparison of the numbers of glycans, glycosites, and glycoproteins in upregulated and downregulated glycopeptides. (**F**) Analysis comparing changes in glycopeptides with changes at the total protein level in aged ovaries. (**G**) Representative Gene Ontology (GO) terms associated with up- and down-regulated glycopeptides, showing enriched biological processes. (**H** and **I**) Proportion of glycan subtypes, core structures, and branch structures in upregulated (H) and downregulated (I) glycopeptides, associated with specific biological processes. The percentages were calculated based on the differential glycopeptides modified by each core or branch glycan structure. Blue and purple text shows the number of glycoproteins and glycopeptides, respectively. (**J** and **K**) Distribution of core (J) and branch (K) glycan structures in glycopeptides related to reactive oxygen species (ROS) biosynthetic and metabolic processes, emphasizing structural changes linked to ROS regulation. (**L**) Top 10 glycan structures with the most significant changes in modified glycosites. Percentages calculated as number of changes divided by total number. (**M**) Comparative analysis of core- and antenna-fucosylated *N*-glycans, focusing on age-related alterations. (**N**) Enrichment of biological processes in glycoproteins solely modified by core-fucosylated (CF) and antenna-fucosylated (AF) *N*-glycans. Data presented as mean ± s.e.m. ∗*P* < 0.05, ∗∗*P* < 0.01, ∗∗∗*P* < 0.005 by two-tailed *t*-test (D).

To investigate glycosylation changes during ovary aging, we performed TMT-based quantification of glycopeptides between two age groups. Principal component analysis (PCA) showed distinct clustering along the PCA1 axis, indicating considerable differences in glycopeptides between young and aged ovaries (Fig. 3A). By using the *p*-value<0.05 and FC > 1.5 as a reasonable cutoff, we identified 1556 up- and 293 down-regulated glycopeptides in aged ovaries (Fig. 3B and C; Table S4). The overall increase of glycosylation with aging detected by mass spectrometry was further confirmed by the Periodic Acid-Schiff (PAS) staining, which clearly showed a heavy layer of glycan accumulation in both follicle and non-follicle regions of aged ovaries (Fig. 3D). We also analyzed the expressions of key OST components (STT3A, STT3B, RPN1, RPN2, etc.) using our global proteomic data. Although some OST subunits exhibited modest upregulation in aged ovaries, these changes did not reach statistical significance (Fig. S5C). The up-regulated glycopeptides were comprised of 273 *N*-glycans and 521 glycosites from 365 glycoproteins, while down-regulated glycopeptides were modified by 180 glycans at 96 glycosites from 87 glycoproteins (Fig. 3E). Clearly, the up-regulated glycopeptides typically exhibited single glycan changes across multiple glycosites, whereas down-regulated glycopeptides showed multiple glycans alterations at each glycosite.

To determine whether these glycopeptide changes occurred at the protein expression or glycosylation level, we performed an integrated analyses on proteomic and glycoproteomic data. At the global proteome level, only 2 % of 9033 quantified proteins exhibited an at least 1.5-fold change in aged ovaries, whereas 11 % of intact glycopeptides exceeded this threshold. Among 381 glycoproteins that contained differential glycopeptides between two age groups, only 49 glycoproteins (12.9 %) were altered at the protein expression level, the remaining 83 % were changed at the glycosylation level (Fig. 3F). These data indicated that aging-related glycopeptide changes largely occurred at the site-specific glycosylation level. To acquire the potential functions of glycoproteins with altered site-specific glycans during ovarian aging, we performed Gene Ontology analysis (GO) on these glycoproteins. The glycoproteins with up-regulated glycans were mainly involved in reactive oxygen species (ROS) biosynthetic, collagen metabolic, and immune system process. While the glycoproteins with down-regulated glycans were mainly involved in the biological processes of ROS metabolic, embryo development, sperm-egg recognition, and reproductive process (Fig. 3G). By further comparing the glycan structures among glycoproteins associated with different biological processes, we found that the collagen metabolism process preferred to have a much higher proportion of Lewis^x/a^ structures, along with less hybrid as well as core-fucosylated glycans compared to other biological processes involved by the up-regulated glycopeptides (Fig. 3H). Glycoproteins involved in sperm-egg recognition and the reproduction process, in which most decreased glycopeptides were actually caused by their down-regulated protein expression in aged ovaries, exhibited higher proportions of complex glycans, fucosylated core structure, as well as LacdiNAc-containing branch structures compared to glycoproteins associated with other biological processes (Fig. 3I).

Especially, the enhanced ROS biosynthetic and declined metabolic processes preferred to have different core and branch structure changes, and the related glycopeptide changes mainly occurred at the glycosylation level instead of protein expression level (these two terms could not be enriched by the differential proteome). Specifically, the increased glycopeptides related to ROS biosynthetic process characterized by more typical core structures (69 %) but less fucosylated core structure (28 %), whereas the decreased glycopeptides associated with ROS metabolic process had less typical core structures (29 %) but more fucosylated core structure (71 %) (Fig. 3J). All above data indicated that the proportion of the fucosylated core structure was decreased in both ROS biosynthetic and metabolic processes in aged ovaries, implying the potential roles of core fucosylation in ROS related processes during ovarian aging [29]. At the branch structure level, two branches NHG (38.3 %) and N(S)H (1.7 %) only existed in the increased glycopeptides involved in the ROS biosynthetic process, while two other branch structures, the sole HexNAc (30 % vs 6.7 %) and LacNAc (40 % vs 21.7 %), showed more abundances in decreased glycopeptides associated with the ROS metabolic process (Fig. 3K).

### 2.5 Core-fucosylation display high-frequency alterations

Based on the number of modified glycosites (excluding oligo-mannose glycans), all top ten up-regulated glycans in aged ovaries were sialylated by Neu5Ac or Neu5Gc, with eight were also core-fucosylated. While among top ten down-regulated glycans in aged ovaries, eight were core-fucosylated, whereas only four were sialylated (Fig. 3L). These results indicated that fucosylation changed at a high-frequency at both directions, while sialylation was mainly increased with ovarian aging. Further analyses showed that over 51 % solely core-fucosylated, 24 % solely antenna-fucosylated, and 40 % dual-fucosylated glycans were changed with ovarian aging (Fig. 3M). Especially, the solely core- and antenna-fucosylated glycoproteins associated with aging preferred to participate in different biological processes. Solely core-fucosylated *N*-glycoproteins were mainly involved in cell adhesion, defense response, response to stress, immune response, acute inflammatory response, cell surface receptor signaling pathway, and blood vessel development, while solely antenna-fucosylated *N*-glycoproteins primarily regulated metabolic, catalytic, and various hydrolase activities (Fig. 3N).

### 2.6 LacdiNAc glycans mainly decrease with the zona pellucida decline

**Fig. 4:**
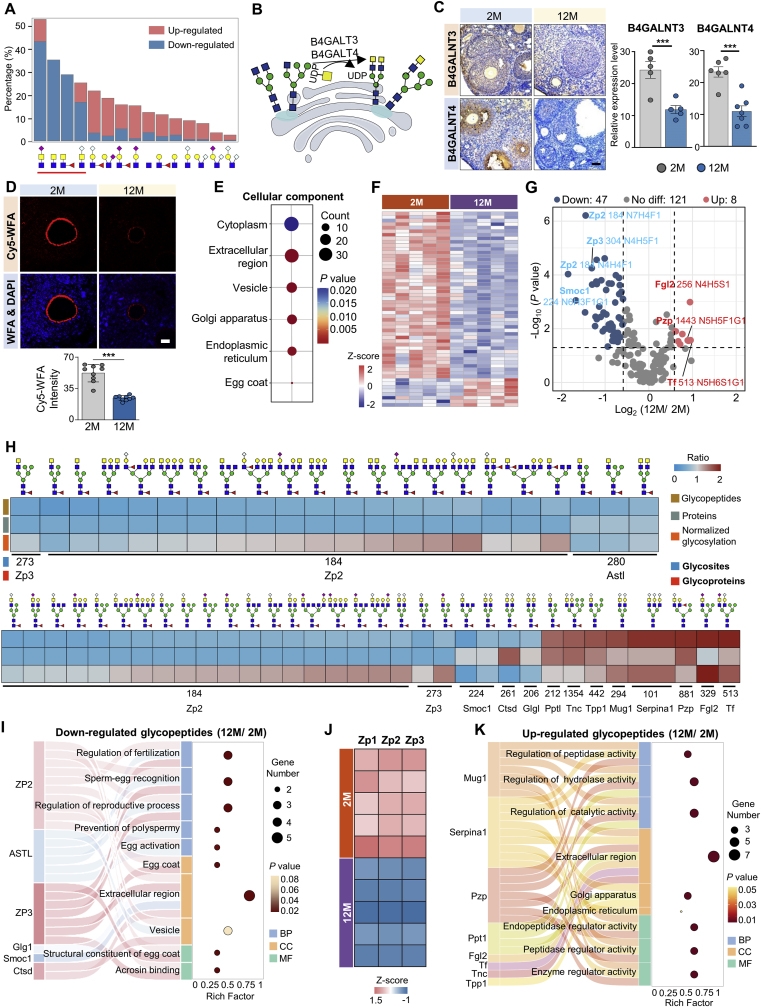
LacdiNAc glycans decrease mainly with zona pellucida decline. (**A**) Proportion of changed glycans containing each structural feature to the total numbers of glycans containing the same glycan feature. (**B**) Schematic of LacdiNAc synthesis pathways. (**C**) Immunohistochemistry of B4GALNT3 and B4GALNT4 protein expressions in mouse ovarian tissues. Scale bar, 50 μm. (**D**) Lectin histochemistry analyses of mouse ovarian tissues based on the Cy5-labeled WFA staining. Scale bar, 50 μm (**E**) Subcellular localization of all identified glycoproteins contaning LacdiNAc glycans. (**F**) Heatmap displaying differentially expressed LacdiNAc-containing *N*-glycopeptides. (**G**) Volcano plot showing upregulated and downregulated glycopeptides with LacdiNAc glycans in aged ovaries. (**H**) Heatmap of altered LacdiNAc-modified glycopeptides, showing the fold changes in the expression of intact glycopeptides (upper row), global proteind (middle row), and normalized glycosylation (bottom row) in aged ovaries. (**I**) Sankey diagram highlighting representative GO terms for downregulated LacdiNAc-containing glycoproteins. (**J**) Expression levels of zona pellucida proteins (ZP1, ZP2, ZP3) in mouse ovaries. (**K**) Sankey diagram showing representative GO terms for upregulated LacdiNAc-containing glycoproteins. Data presented as mean ± s.e.m. ∗*P* < 0.05, ∗∗*P* < 0.01, ∗∗∗*P* < 0.005 by two-tailed *t*-test (C and D).

We also calculated the proportion of changed glycans with each structural feature to their total numbers (based on their modified glycosites), and found that all top four changed branch structures were LacdiNAc-containing branches, with the majority showing a distinct decline in aged ovaries (Fig. 4A). The overall decrease of LacdiNAc structures in aged ovaries was first verified by the reduced expression of the corresponding β1-4-N-Acetyl-Galactosaminyltransferase 3 (B4GN3) and β1-4-N-Acetyl-Galactosaminyltransferase 4 (B4GN4), which were measured by the immunohistochemistry assay (Fig. 4B and C). The immunohistochemistry results indicated that both B4GN3 and B4GN4 were mainly located surrounding the follicles in young ovaries but dramatically reduced in middle-aged ovaries. The overall decrease of LacdiNAc structures in aged ovaries was also confirmed by the lectin histochemistry using Cy5-labeled Wisteria floribunda agglutinin (WFA), which specifically binds LacdiNAc glycans [30]. The histochemical results revealed a noticeable decrease in WFA staining in the zona pellucida with a wrinkled morphology in aged ovaries (Fig. 4D). A total of 176 LacdiNAc-containing glycopeptides were identified from 50 glycoproteins in mouse ovary (Table S5), which were mainly located at the cytoplasm, extracellular region, vesicle, Golgi apparatus, endoplasmic reticulum and egg coat (zona pellucida) (Fig. 4E). By using *p*-value<0.05, FC > 1.5 as a cutoff, we identified 55 changed glycopeptides from 14 glycoproteins, of which 8 glycopeptides from 8 glycoproteins were up-regulated and 47 glycopeptides from 6 glycoproteins were down-regulated in aged ovaries (Fig. 4F and G).

By integrating with quantitative proteome data, we found that the majority of decreased LacdiNAc-containing glycopeptides (40/47) were identified from the zona pellucida sperm-binding protein 2 (ZP2) and zona pellucida sperm-binding protein 3 (ZP3), which were actually decreased at the protein expression level (Fig. 4H). In addition, the astacin-like metalloendopeptidase (ASTL) that contained 3 decreased LacdiNAc-containing glycopeptides also showed a 0.75-fold decrease with aging. All these three glycoproteins were located at the zona pellucida and participated in the biological processes of egg activation [31], sperm-egg recognition and binding [32], prevention of polyspermy [33], and etc (Fig. 4I). In addition, the zona pellucida sperm-binding protein 1 (ZP1), which was also modified by a LacdiNAc glycan at the glycosite Asn-49 (the glycopeptide didn't show significant change), was also significantly decreased at the protein expression level in aged ovaries (Fig. 4J; Table S5). Collectively, these findings suggested that the observed reduction in zona pellucida glycoproteins and LacdiNAc glycans may be attributed to the decline in follicle numbers in aged ovaries. To facilitate the future female reproduction studies, the site-specific *N*-glycan mapping of ZP1, ZP2 and ZP3 have been summarized in Fig. S6. The high percentages of LacdiNAc glycans attached at three zona pellucida-related glycoproteins implied the important roles of LacdiNAc in zona pellucida and female fertility.

In contrast, most up-regulated LacdiNAc-containing glycopeptides increased at the glycosylation level (6/8) (Fig. 4H). The cellular localization of the related glycoproteins, which are predominantly located in the Golgi and extracellular regions, differs from the above glycoproteins with down-regulated LacdiNAc glycopeptides in aged ovaries. Among 8 glycoproteins containing up-regulated LacdiNAc-containing glycopeptides, 4 of them including the murinoglobulin-1 (MUG1), alpha-1-antitrypsin 1 (AAT1), pregnancy zone protein (PZP), and palmitoyl-protein thioesterase 1 (PPT1) were mainly involved in the regulation of various enzyme activities during ovarian aging (Fig. 4K).

### 2.7 Elevated Neu5Ac and Neu5Gc play distinctive immune regulation roles

**Fig. 5:**
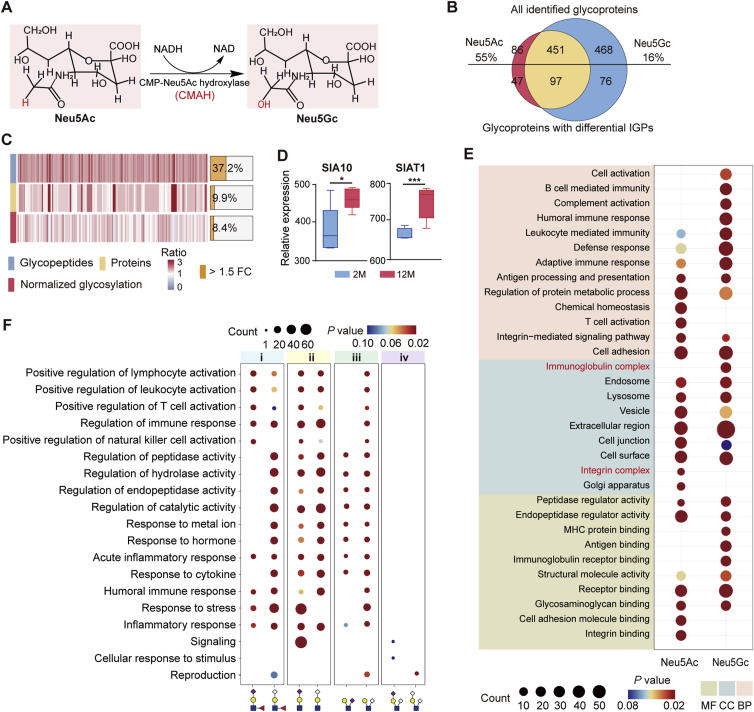
**Distinct immune regulatory roles of increased Neu5Ac and Neu5Gc in ovarian aging.** (**A**) Conversion process of CMP-Neu5Ac to CMP-Neu5Gc by the Cytidine Monophospho-N-Acetylneuraminic Acid hydroxylase (CMAH). (**B**) Comparison of glycoproteins modified with *N*-glycans containing Neu5Ac and Neu5Gc in all (upper) and differential glycoproteins (bottom). (**C**) Heatmap showing increased sialic acid-containing *N*-glycopeptides, indicating that a large number of sialylated *N*-glycopeptides were changed at glycosylation level instead of protein expression level in the 12-month-old mouse ovaries (left). Stacked chart displaying the proportions of differential glycopeptides, proteins or normalized glycosylation (right). (**D**) The protein expression levels of sialic acid-related glycosyltransferases, ST3 Beta-Galactoside Alpha-2,3-Sialyltransferase 6 (SIA10) and ST6 Beta-Galactoside Alpha-2,6-Sialyltransferase 1 (SIAT1). (**E**) Comparison of enriched biological processes, cellular components and molecular functions between glycoproteins solely modified with Neu5Ac and Neu5Gc. (**F**) Representative biological process terms for four categories (i-iv) of sialylated *N*-glycopeptides, representing eight types of sialylated branch structures.

N-acetylneuraminic acid (Neu5Ac) and N-glycosylneuraminic acid (Neu5Gc) are two major types of sialic acids, differing by only one oxygen atom (Fig. 5A). Although nearly half of the Neu5Gc-containing glycans and most Neu5Ac-containing glycans were found on the same glycoproteins, we did identify 468 and 86 glycoproteins that were only modified by Neu5Gc and Neu5Ac, respectively (Fig. 5B). Notably, 55 % and 16 % of glycoproteins solely modified by Neu5Ac and Neu5Gc exhibited an at least 1.5-fold change in aged ovaries, respectively. Integrated glycoproteomic and proteomic analyses revealed that the majority of these sialylated glycopeptides were increased at glycosylation level (Fig. 5C). The overall increase of sialylation was also confirmed by the upregulated ST3 β-galactoside ɑ-2,3-sialyltransferase 6 (SIA10) and ST6 β-galactoside ɑ-2,6-sialyltransferase 1 (SIAT1) that were responsible for sialylation based on the proteomic data (Fig. 5D). Interestingly, we found that glycoproteins solely modified by increased Neu5Gc and Neu5Ac *N*-glycans with aging preferred to perform different biological functions. Specifically, 76 glycoproteins modified solely by Neu5Gc were located at immunoglobulin complex and involved in immune response, complement activation, and defense response. In contrast, 47 glycoproteins uniquely modified by Neu5Ac were primary located in the integrin complex, playing roles in the integrin-mediated signaling pathway and T cell activation (Fig. 5E).

The detailed structures of sialylated branches might also affect the functions of glycans and related glycoproteins. Apart from sialylated LacdiNAc that have been analyzed above, we classified all other significantly altered sialoglycopeptides into four groups (Fig. 5F; Table S6). Among glycoproteins modified by sialyl Lewis^x/a^ (Fig. 5F–i), those with Neu5Ac were primarily involved in the positive regulation of immune cell activation including lymphocytes and leukocytes, while those with Neu5Gc mainly regulated various enzyme activities such as peptidases and hydrolases**.** Among glycoproteins modified by terminal-sialylated LacNAc (Fig. 5F–ii), although most GO categories were overlapped between two groups, those terminated with Neu5Ac were solely involved in the signaling and response to stress. As for glycoproteins modified by two types of antenna-sialylated LacNAc glycans (Fig. 5F–iii), although they were commonly involved in the regulation of various enzyme activities, these with antenna-Neu5Gc LacNAc were also involved in the regulation of various immune cells. As for the bi-sialyl LacNac glycans (Fig. 5F–iv), these with Neu5Gc played crucial roles in reproductive processes. All above results suggested that not only the types of sialic acids but also the detailed structures of sialylated branches may affect the immune roles of their modified glycoproteins.

### 2.8 Glycosylation change involved in signaling pathways

**Fig. 6:**
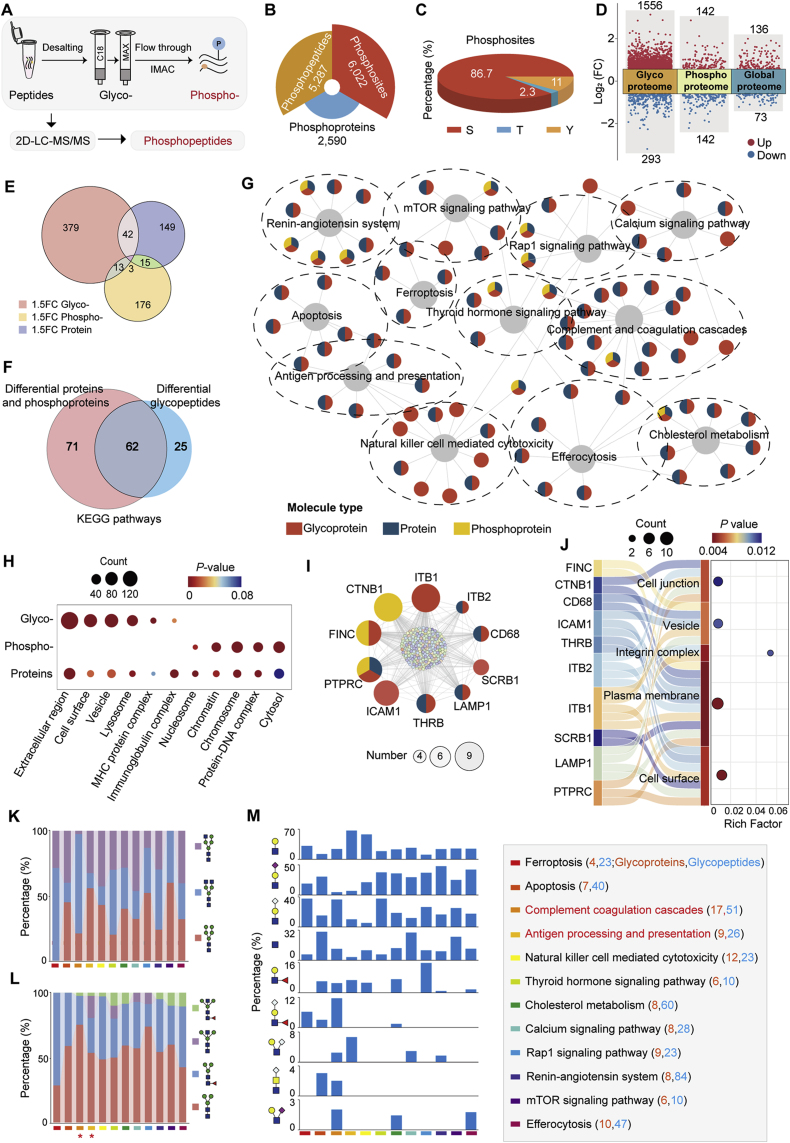
**Different roles of glycosylation and phosphorylation in signaling pathways.** (**A**) Workflow of phosphoproteomic analysis. (**B**) Summary of identified phosphopeptides, phosphosites and phosphoproteins in mouse ovaries. (**C**) Distribution of identified phosphorylation sites. (**D**) Volcano plot showing up- and down-regulated glycopeptides, phosphopeptides, and proteins in aged ovaries. (**E**) Comparing differential glycoproteins, phosphoproteins, and proteins in young and aged ovaries. (**F**) Venn diagrams of shared and unique KEGG pathways for differentially expressed glycopeptides (DEGPs) and differential proteins (including proteins and phosphoproteins). (**G**) Annotation of molecules of PPI network associated with the ovarian aging pathways derived from shared KEGG pathways. (**H**) Subcellular localization of differentially expressed glycoproteins, phosphoproteins, and proteins. (**I**) Top 10 hub proteins identified in the PPI network of co-enriched pathways involving DEGPs and differential proteins (including proteins and phosphoproteins). (**J**) Subcellular localization of glycoproteins among the top 10 hub proteins. (**K** to **M**) Comparison of glycan subtypes (K), core structures (L), and branch structures (M) on glycoproteins associated with different KEGG pathways. Red and blue text represent the number of glycoproteins and glycopeptides, respectively. DEGPs, differentially expressed glycopeptides. KEGG, Kyoto Encyclopedia of Genes and Genomes. PPI, protein-protein interaction.

To further explore how glycosylation changes affect the cellular phenotypes of ovaries, we performed signaling pathway analyses on these altered glycopeptides/glycoproteins by integrating quantitatively phosphoproteomic and proteomic data. From the same TMT-labeled samples, we identified 5287 unique phosphopeptides containing 6022 phosphosites from 2554 phosphoproteins (Fig. 6A and B; Table S7). Among these phosphosites, 86.7 % were phosphoserine (pS), other 11 % and 2.3 % were phosphothreonine (pT) and phosphotyrosine (pY), respectively (Fig. 6C). These were consistent with previously reported phosphorylation site distributions [34]. In aged ovaries, 142 phosphopeptides were significantly up-regulated and 142 were down-regulated (*p*-value<0.05, FC > 1.5**)**. Additionally, the proteomic data identified 36 up-regulated and 73 down-regulated proteins (*p*-value<0.05, FC > 1.5. Fig. 6D and E; Table S7). Integrated pathway analyses revealed that a total of 62 pathways were co-enriched among altered phosphoproteins, glycoproteins, and proteins. Notably, the glycoproteins with altered site-specific glycans in aged ovaries were involved in nearly half of the pathways enriched by differential phosphoproteins and proteins (Fig. 6F). These commonly involved pathways included the complement and coagulation cascade, antigen processing and presentation, ferroptosis, and apoptosis, which were associated with reduced fertility and a disturbed immune microenvironment commonly occurring during ovarian aging (Fig. 6G). Especially, these aging-related glycoproteins that were enriched in various signaling pathways were mainly located on the cell surface, while differential phosphorylated proteins were mainly located in the cytosol and nucleus (Fig. 6H), suggesting that altered glycans were located at the upstream of the related signaling pathways compared to phosphorylation.

We also performed protein-protein interaction (PPI) analyses on glyco-, phospho- and global proteins that were commonly enriched in various pathways. The interconnection existed within different pathways underscored the complexity of signaling pathway regulation during ovarian aging (Fig. 6G). CytoHubba analysis by combining 12 algorithms revealed that nine of the top ten hub proteins were glycoproteins (Fig. 6I), with mostly located on the cell surface or plasma membrane, forming integrin complexes and participating in cellular junction (Fig. 6J). Interestingly, we found that different glycan changes were involved in different signaling pathways. For example, when comparing the altered glycan features of the complement and coagulation cascade with the antigen processing and presentation, we found that altered glycans involved in the complement coagulation cascades were featured by a high proportion of complex glycans (76.5 %), while altered glycans involved in the antigen processing and presentation were largely oligo-mannose glycans (55.8 %) (Fig. 6K). As for the core structures, the bisected core structure was notably prevalent in altered glycans related to antigen processing and presentation (17.1 %), but were absolutely absent in altered glycans associated with the complement coagulation cascades (Fig. 6L). In addition, the complement coagulation cascades pathway had three feature branch structures including the sialylated LacdiNAc, antenna-Neu5Ac LacNAc, and terminal-Neu5Gc lewis^x/a^, whereas the antigen processing and presentation pathway had more LacNAc and antenna-Neu5Gc LacNAc (Fig. 6M). The above observations again implied the important roles of different sialoglycans in regulating aging-related immune processes (as shown in Fig. 5).

**Fig. 7:**
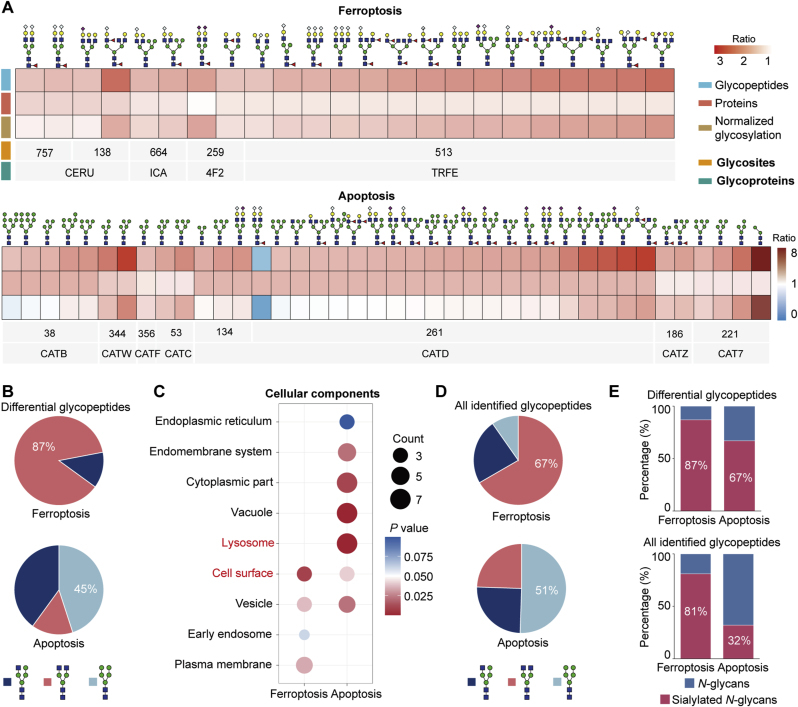
**Distinct glycoforms associated with ferroptosis and apoptosis.** (**A**) Heatmap showing ferroptosis- and apoptosis-associated differentially expressed glycopeptides, mostly increased at the glycosylation level rather than at the protein expression level. (**B**) Distribution of glycan subtypes in differential glycopeptides associated with ferroptosis and apoptosis. (**C**) Cellular components of differential glycoproteins associated with ferroptosis and apoptosis. (**D**) Distribution of glycan subtypes on all glycopeptides associated with ferroptosis and apoptosis. (**E**) Distribution of sialylated *N*-glycans on differential or all glycopeptides associated with ferroptosis and apoptosis.

During the pathway analyses, we also found that both ferroptosis and apoptosis were enriched by upregulated glycopeptides, indicating the crucial roles of these two distinct cell death types in ovarian aging [35,36]. Further analyses showed these glycopeptides mainly increased at the glycosylation level rather than the protein expression levels, but the glycopeptides associated with ferroptosis and apoptosis were modified by different glycan structures (Fig. 7A). The differential glycopeptides linked to ferroptosis predominantly featured by complex glycans (87 %), whereas those involved in the apoptosis mainly displayed oligo-mannose glycans (45 %) (Fig. 7B). Cellular localization analyses showed that all apoptosis-associated glycoproteins were located in the lysosome (7/7), whereas the ferroptosis-associated glycoproteins were primarily found on the cell surface (3/4) (Fig. 7C). We then reasoned that the structural differences of changed glycans between ferroptosis and apoptosis might be caused by the cellular localization of glycoproteins involved in these two distinct cell death types. By further analyzing all identified glycopeptides within these two pathways, we confirmed that 67 % of ferroptosis related glycopeptides were complex glycans, whereas 51 % of apoptosis-associated glycopeptides were oligo-mannose glycans (Fig. 7D; Table S8). Besides, a high abundance of sialylated glycopeptides was found in ferroptosis (Fig. 7E). The real roles of glycosylation in regulating different cell death pathways still need further investigation.

## 3 Discussion

Ovarian aging normally precedes the aging of other organ systems and therefore can serves as a pacemaker of female aging [37]. During the last decade, multiple omic approaches such as transcriptomics and proteomics have been widely applied to ovarian aging studies, which definitely give us a better understanding of this complex process [[5], [6], [7]], but it is still far from fully elucidating its molecular mechanisms. Like nucleic acid, protein, and lipid, glycan is also one type of important macromolecule that plays essential roles in various biological processes. It is known that protein glycosylation play integral roles in many essential steps of female reproduction including oocyte development, maturation, sperm-egg recognition, and fertilization [9,10]. It is therefore not unexpected that a large number of important proteins in the female reproductive system are actually glycoproteins [[11], [12], [13]]. However, due to structural complexity of glycans and microheterogeneity of glycosylation, site-specific glycoproteomic analyses on complex samples have just become achievable in recent years [[16], [17], [18], [19], [20], [21], [22]].

In this study, we present the first site-specific and structural glycoproteome landscape of aging mouse ovary by using our StrucGP based glycoproteomic approaches. By linking each site-specific glycan to its subcellular localization, we revealed distinct *N*-glycan signatures across different cellular components. Especially, *N*-glycans at the egg coat were characterized by high proportions of complex glycans, core fucosylation, and LacdiNAc branches, which were different from glycans in other cellular components. Based on TMT-labeling quantification, we further uncovered three major *N*-glycan alterations during ovarian aging, including: 1) frequently changed core-fucosylation that predominantly linked to cell adhesion as well as immune- and stress-related responses, differing from altered antenna-fucosylation that was mainly responsible for various enzyme regulations; 2) remarkably reduced LacdiNAc glycans that were highly enriched on zona pellucida (ZP) glycoproteins; and 3) highly increased sialoglycans modified by Neu5Ac and Neu5Gc that modulated immune activation and responses, respectively. With integrated multi-omics analyses, we also revealed the distinct roles of extracellular and cell surface glycoproteins as the upstream of signaling pathways compared to phosphoproteins that were mainly located in cytosol and nucleus. In addition, comparative analyses of aging-related glycan structures among different biological processes or pathways indicated that the antigen processing and presentation as well as the complement coagulation cascades were featured by high proportion of oligo-mannose and complex glycans, respectively; both ROS biosynthetic and metabolic processes exhibited decreased core-fucosylation but different branch structure changes; and up-regulated glycopeptides associated with ferroptosis and apoptosis showed extensive sialylated and oligo-mannose *N*-glycans, respectively. Altogether, these observations offer a novel glycobiological perspective on ovarian aging, significantly expanding our understanding of its molecular mechanisms beyond other multiple omics analyses such as transcriptomics and proteomics [6,7].

It should be mentioned that when controlling the glycoproteome data only based on the FDR<1 % and at least 5 b/y ions at the glycosite-containing peptide level, we achieved precise identification of 3746 *N*-glycoproteins and 5857 *N*-glycosites from mouse ovaries. The presence of oxonium ions and Y1–Y5 patterns for the core structures of *N*-glycans in all spectra dramatically enhanced the confidence in identifying *N*-glycosites and glycoproteins at the intact glycopeptide level, surpassing the reliability of previous reports that relied solely on de-glycosylated peptides [38,39]. Considering that the ovary expresses certain types of proteins, and only those located at the cell surface, extracellular matrix, and organelles such as the endoplasmic reticulum, Golgi apparatus, and lysosome can undergo N-linked glycosylation, the data presented here should have achieved an in-depth mapping of site-specific *N*-glycans on mouse ovary.

LacdiNAc-containing *N*-glycans were highly enriched on ZP (ZP1, ZP2, and ZP3) and ASTL glycoproteins, which were all declined with ovarian aging. Given that oocyte numbers decreased dramatically during ovarian aging, the reduced expressions of these *N*-glycans might also be a consequence of age-related oocyte loss. ASTL is an ovastacin enzyme that is responsible for cleaving ZP2 to prevent polyspermy and has been linked to female infertility [40]. ZP1, ZP2, and ZP3, integral components of the zona pellucida in mouse ovaries, are also critical for oocytes growth and sperm-egg recognition. ZP1 is thought to cross-link ZP2-ZP3 heterodimers, providing structural stability and integrity to the ZP matrix [41], while ZP2 functions as a secondary sperm receptor, binding acrosome-reacted spermatozoa [42]. ZP3, acting as the primary sperm receptor, binds to acrosome-intact spermatozoa and induces the acrosome reaction [43]. The carbohydrate residues in the ZPs, including sialylated, mannose-rich, and galactose-terminal glycans [[44], [45], [46]], play essential roles in sperm-egg interaction. All those reported glycans were also identified in this study. LacdiNAc-containing glycans, although rarely exist on mammalian glycoproteins, have previously reported to have profound effects on the regulation of the ovulatory cycle [47], suggesting their important toles in female reproduction. The StrucGP software successfully identified these uncommon LacdiNAc-containing *N*-glycans at the glycopeptide level based on their feature B ions complementary with the related Y ions, and therefore overcome the limitations of other methods in recognizing these structural features [16,48]. Our study not only provides the most comprehensive site-specific glycan map of ZP glycoproteins but also offers novel potential targets for improving ovarian functions in middle-aged females.

Sialylated glycans exhibited another notable age-related change, with generally increasing at the glycopeptide level rather than the protein expression level. Previous research has highlighted the essential roles of sialylated *N*-glycans in embryogenesis, development, cancer and immune processes [49,50]. It should be noted that the increased infiltration of immune cells with age might also be a critical factor that could contribute to the changes in glycan patterns [51,52]. However, the distinct functions of N-acetylneuraminic acid (Neu5Ac) and N-acetylneuraminic acid (Neu5Gc) in immune process remain unclear. Our data revealed that Neu5Ac/Gc-modified glycoproteins preferred to perform distinct functions. Specifically, Neu5Ac-modified glycoproteins were predominantly associated with the integrin complex, playing roles in integrin-mediated signaling pathways and T cell activation. In contrast, Neu5Gc-modified glycoproteins were mainly located in the immunoglobulin complex, participating in immune response, complement activation, and defense response. Even though most up-regulated sialoglycans were linked to immunity, it appeared that Neu5Ac-modified glycoproteins were altered prior to Neu5Gc-modified glycoproteins in this important process. In addition, our data suggested that functional differences of Neu5Ac and Neu5c also existed within different types of branch structures, implying the possibilities of sialoglycan patterns as potential indicators of ovarian immunity status.

The real role of protein glycosylation changes associated with ovarian aging has not been extensively studied. The site-specific and structural glycoproteomic data presented here facilitated the molecular network analyses and functional studies by easily linking each altered glycan structure to its modified glycosite and glycoprotein. Based on our results, the differential glycopeptides in aged ovaries were predominantly located at the extracellular regions and cell surface [53], and the involved signaling pathways including antigen processing and presentation, complement cascades [7], cholesterol metabolism [54], ferroptosis [55], and apoptosis [6], were all associated with impaired ovarian reserve and functional decline [52]. Considering almost all phosphoproteins were found in the cytosol and nucleus [56], the glycoproteins might be located at the upstream of various signaling pathways compared to phosphoproteins. Especially, although both ferroptosis and apoptosis have been extensively studied in ovarian aging, we provided the first glycoproteomic evidence that there were significant differences in *N*-glycan structural changes existed within these two types of cell death. Specifically, ferroptosis-associated glycoproteins were exclusively sialylated, while apoptosis-associated glycoproteins predominantly contain oligo-mannose glycans. The upregulated sialoglycans might be able to promote iron uptaking to enhance aging-related ferroptosis through a series of signaling pathways, such as a previously reported exogenous (or transporter-dependent) pathway [57]. Those findings open up new possibilities for considering *N*-glycan features as potential markers to differentiate these two or even more types of cell death.

We realize that additional evidences are still required to confirm these site-specific glycosylation changes associated with ovarian aging. Although the mouse remains the predominant animal model for ovarian aging as well as other human-related studies, intrinsic differences should not be ignored among mice, nonhuman primates, and humans. Especially, humans express only Neu5Ac but not Neu5Gc, suggesting that sialoglycan alterations observed here are unable to directly transfer to the studies of human ovarian aging. Eventually, molecular biology approaches are also essential to elucidate the underlying mechanisms of glyco-alterations on ovarian aging. Also, the orthogonal validation is also necessary before further function or mechanistic studies, as the accurate assignment of each specific glycan structure only based on the MS/MS spectra remains challenging. Despite of these limitations, these findings provide a solid foundation for future functional and mechanical studies of ovarian aging from a glycobiological perspective.

## 4 Methods

### 4.1 Mouse ovary collection

Female C57BL/6J mice were obtained from Huachuang Xinnuo Pharmaceutical Technology Co. (Jiangsu, China) at young (2-month-old, 18.27 ± 0.57g, n = 15) and middle age (12-month-old, 32.40 ± 1.74g, n = 15). All mice were maintained under a 12:12-h light/dark cycle at a constant temperature (25 °C) with humidity levels between 35 % and 75 %. The mice were anesthetized with intraperitoneal injection of chloral hydrate (0.6 mg/g body weight), and cardiac perfusion was performed to minimize blood interference in subsequent experiments. One ovary from each of three randomly selected mice in each age group was collected and fixed for paraffin embedding, while all remaining ovarian tissues were collected from all mice and frozen in liquid nitrogen before being stored at −80 °C until use. All procedures complied with ethical regulations and were approved by the Ethics Committee of Northwest University, China (Approval No. NWU-AWC-20240901 M).

### 4.2 Histology

The ovaries from a total of six mice as mentioned above were fixed, paraffin-embedded, and sectioned at 5 μm thickness. Every fifth sections were selected for follicle counting using hematoxylin and eosin (H&E) staining (cat. no. YK2222, Y&K Bio, China). Follicles in histologic sections were classified as primordial, primary, secondary, and antral follicles based on the morphology of surrounding somatic cells. Atretic preantral follicles were recognized according to morphologic criteria previously reported for consistency in comparison and evaluation [58,59].

### 4.3 Masson's Trichrome and Sirius Red staining

Collagen contents were assessed using Masson's Trichrome (cat. no. YK2223, Y&K Bio, China) and Sirius Red staining kits (cat. no. YK2224, Y&K Bio, China) following the manufacturer's instructions. In brief, ovarian paraffin sections were deparaffinized in xylene, dehydrated in different concentrations of ethanol, and stained with Masson's Trichrome and Sirius Red. Collagen fibers stained red in Sirius Red and blue in Masson's Trichrome. The sections were observed under an automatic digital slide scanning imaging system (slidescan, China) and quantified using Image J software (v.1.52).

### 4.4 Periodic Acid-Schiff staining

Glycogen detection was performed with a Periodic Acid-Schiff (PAS) assay kit (cat. no. YK2206, Y&K Bio, China) according to the manufacturer's instructions. Periodic acid oxidation converted intracellular polysaccharides to a dialdehydes, which reacted with Schiff's reagent to produce a red coloration. The sections were scanned with an automatic digital slide scanning imaging system (slidescan, China) and quantified using Image J software (v.1.52).

### 4.5 Immunohistochemistry

Immunohistochemistry was performed as previously described [60]. Briefly, pretreated ovarian tissue sections were blocked and incubated with an anti-B4GALNT3 antibody (1:100, cat. no. orb317451, Biorbyt, UK) or anti-B4GALNT4 antibody (1:1000, cat. no. orb546266, Biorbyt, UK) overnight with shaking at 4 °C. The secondary antibody reagent was then added to completely cover the tissue, and incubated at room temperature for 20 min. Staining was visualized using a DAB chromogenic kit (cat. no.DAB-1031, MXB, China). The sections were observed under an automatic digital slide scanning imaging system (slidescan, China), and 5 different fields of view were randomly selected in each section for quantification.

### 4.6 Fluorescence-based lectin histochemistry

Fluorescence-based lectin histochemistry was performed to analyze LacdiNAc structures in mouse ovarian tissues. Formalin-fixed paraffin-embedded (FFPE) tissue sections were subjected to dewaxing using xylene, following by rehydration through a graded alcohol series. The staining procedure was conducted following established protocols [60] with minor modifications. Briefly, tissue sections were washed with phosphate-buffered saline (PBS), and then blocked with 5 % (w/v) bovine serum albumin (BSA) containing 0.04 % Triton X-100 for 1h at room temperature (RT). Subsequently, sections were incubated with 1ug/μl Cy5-labeled WFA overnight in the dark at RT, followed by staining with DAPI (cat. no. P0131, Beyotime, China). Fluorescence imaging was performed uaing a confocal microscopy, and quantitative analysis of fluorescence intensity was conducted using Image J software (v.1.52).

### 4.7 Protein extraction and trypsin digestion

Each ovarian tissue was cut into around 1 mm^2^ fragments and washed three times with precooled phosphate-buffered saline (PBS, pH 7.4). Subsequently, the ovarian tissues were denatured in an 8 M urea (cat. no. U5128, Sigma, Germany)/1 M NH_4_HCO_3_ (cat. no. 11213, Sigma, Germany) solution, homogenized through physical grinding with precooled steel balls using an automatic rapid tissue homogenizer (Shanghai Jing Xin, China) at 60 Hz for 2 min, and sonicated until clear. Lysates were centrifuged at 15,000 g for 15 min at room temperature, and protein concentrations in the supernatants were measured using a BCA protein assay reagent (cat. no. P0012, Beyotime, China). Due to the limited tissue size, proteins from three mice were pooled to form five biological replicates per age group. Proteins were reduced with 5 mM dithiothreitol (DTT) (cat. no. 43819, Sigma, Germany) at 37 °C for 1 h, followed by alkylation with 15 mM iodoacetamide (IAM) (cat. no. I6125, Sigma, Germany) at room temperature (RT) in the dark for 30 min. Another 2.5 mM DTT was then added and incubated at RT for 10 min. The solutions were diluted twofold with deionized water prior to the first cycle of protein digestion by incubating the samples with the sequencing grade trypsin (cat. no. V5113, Promega, USA, proteins: enzyme, 100:1, w/w) for 2 h at 37 °C with shaking. Samples were further diluted fourfold with deionized water and an equivalent amount of trypsin (proteins: enzyme, 100:1, w/w) was added for overnight digestion at 37 °C with shaking. The digested samples were acidified with 10 % trifluoroacetic acid (TFA) (cat. no. 3020031, Sigma, Germany) to pH < 2, centrifuged at 15,000 g to remove any particulate matters, and desalted using C18 columns (cat. no. WAT054955, Waters, USA). Peptides were eluted with 60 % acetonitrile (ACN) (cat. no. A9554, Thermo Fisher Scientific, USA)/0.1 % TFA solution and the peptide concentrations were measured by the UV absorbance at 215 nm using a DS-11 spectrophotometer (DeNovix, USA).

### 4.8 Tandem mass tag (TMT) labeling

Equal amount of tryptic peptides from each pooled sample (five young and five middle-aged) were labeled with one channel of 10-plex TMT reagents following the manufacturer's protocols (cat. no. YA332834, Thermo Fisher Scientific, USA). The TMT channels were listed as fellows: five young pooled samples were labeled by the channels of 126, 127 N, 127C, 128 N, and 128C, respectively. While five middle-aged pooled samples were labeled by the channels of 129 N, 129C, 130 N, 130C, and 131, respectively. The peptides were incubated with TMT reagents overnight at room temperature, and all ten TMT labeled samples were then combined into a single pooled sample and desalted using a C18 column (cat. no. WAT054955, Waters, USA). Approximate 5 % of pooled sample was used directly for global proteomic analysis via 2D-LC-MS/MS, while the remaining 95 % was used for sequential enrichment of glyco- and phospho-peptides for glycoproteomic and phosphoproteomic analyses.

### 4.9 Intact glycopeptide enrichment

Intact glycopeptides were enriched from TMT-labeled pooled peptides using an Oasis Mixed Anion-Exchange (MAX) column [61] (cat. no. 186000366, Waters, USA). Peptides eluted from the C18 column in 60 % ACN/0.1 % TFA were diluted to a final concentration of 95%ACN/1%TFA with 100 % ACN and 10 % TFA solution. The peptide solution was loaded onto the MAX extraction cartridge twice, followed by three washes with 95 % ACN/1 % TFA. Glycopeptides were then eluted in 400 μL of 50 % ACN/0.1 % FA, dried using an RVC 2–18 CDplus concentrator (Christ, Osterode am Harz, Germany). The sample was then resuspended in 20 μL of 0.1 % FA for two-dimensional liquid chromatography - tandem mass spectrometry analysis (2D-LC-MS/MS).

### 4.10 Phosphopeptide enrichment

Phosphopeptides were enriched using immobilized metal affinity chromatography (IMAC) as previously described [62]. Briefly, the Fe^3+^-NTA agarose beads were freshly prepared from Ni^2+^-NTA agarose beads by chelation with 100 mM EDTA and 10 mM FeCl_3_ [63]. Prior to enrichment, the beads were washed three times with HPLC-grade water and resuspended in the buffer (Beads: ACN: Methanol: 0.01 % Acetic Acid = 1: 1: 1: 1). Peptides collected from the flow-through of MAX columns were adjusted to 80 % (v/v) ACN with 0.1 % (v/v) TFA to a final concentration of 0.5 μg/μl and incubated with Fe^3+^-NTA beads for 30 min at RT. Following incubation, the beads were separated from the peptide solution by centrifugation and washed three times with binding/washing buffer. Phosphopeptides were eluted using 500 mM potassium phosphate buffer (KH2PO4, pH 7.0) with 50 % (v/v) ACN in 0.1 % (v/v) formic acid. The eluted phosphopeptides were desalted using C18 (cat. no. 66883-U, Supelco, Germany), then dried and reconstituted in 3 % (v/v) ACN with 0.1 % (v/v) formic acid for downstream analysis.

### 4.11 High-pH HPLC fractionation

Peptides, glycopeptides and phosphopeptides from TMT labeled samples were individually fractionated into 24 fractions on a 1260 Series HPLC system (Agilent Technologies, USA) using a Zorbax Extend-C18 analytical column (1.8 μm particle size) at a flow rate of 0.2 mL/min. The mobile-phase A consisted of 10 mM ammonium formate (pH 10), while mobile-phase B was composed of 10 mM ammonium formate and 90 % ACN (pH 10). Sample separation was accomplished using the following linear gradient: 0–2% B for 10 min, 2–8% B for 5 min, 8–35 % B for 85 min, 35–95 % B for 5 min, 95-95 % B for 15 min. Peptides were detected at 215 nm and 96 fractions were collected along with the LC separation in a time-based mode from 3 to 112 min. The 96 fractions of each sample were combined into 24 fractions. The samples were then dried in a Speed-Vacuum and stored at −80 °C until LC-MS/MS analysis.

### 4.12 LC-MS/MS analysis

For LC-MS/MS analysis, each sample (approximately 1 μg of labeled peptides, phosphopeptides, or intact glycopeptides) was separated on an Orbitrap Fusion Lumos Mass Spectrometer equipped with an Easy-nLCTM 1200 system (Thermo Fisher Scientific, San Jose, CA, USA). Peptide separation was performed on a 75 μm × 50 cm Acclaim PepMap100C18 column (cat. no. 164570, Thermo Fisher Scientific, USA) with a 75 μm × 2 cm pre-column (cat. no. 164946, Thermo Fisher Scientific, USA). The flow rate was maintained at 200 nL min^−1^ using mobile phase A (0.1 % FA in water) and mobile phase B (0.1 % FA in 80 % ACN).

For proteomics and phosphoproteomics, a 130 min gradient was applied as follows: 3–10 % B for 3 min, 10–30 % B for 92 min, 30–68 % B for 8 min, 68–98 % B for 3 min, 98-2% B for 1 min, 2–98 % B for 5 min, 98-2% B for 1 min, and 2–98 % B for 5 min, followed by 2 % B for 12 min. MS1 spectra were acquired in the Orbitrap at 60K resolution across 350–1800 *m*/*z* (AGC 4 × 10^5^). Data-dependent MS/MS was performed using higher-energy collision dissociation (HCD) at a collision energy of 37 % and at 50K resolution, with an isolation width of 1.6 Da. Ions with charges of 2–6 were selected for fragmentation. The spray voltage was set at 2.4 kV.

For glycoproteomics, a 120 min gradient was applied as follows: 3–7% B for 3 min, 7–35 % B for 80 min, 35–68 % B for 8 min, 68–99 % B for 4 min, 99-2% B for 1 min, 2–99 % B for 5 min, 99-2% B for 1 min, and 2–99 % B for 5 min, followed by 2 % B for 13 min. The spray voltage was set at 2.4 kV. MS1 spectra (AGC 4 × 10^5^) were collected from 375 to 1800 *m*/*z* at 50K resolution. Selected precursor ions (charge states of 2–5) were isolated with a *m*/*z* 2 width and subjected to data-dependent HCD fragmentation at 50K resolution. Two MS2 spectra were generated per precursor, one using low-energy HCD (27 %) for glycan structure analysis and another using high-energy HCD (40 %) for peptide sequence identification. Dynamic exclusion was set to 20 s to minimize re-selection of previously fragmented ions.

### 4.13 Database search for proteome and phosphoproteome

All LC-MS/MS data were searched against the reviewed *Mus musculus* reference proteome databases (UniProt, Proteome ID: UP000000589, downloaded on January 11, 2024). Peptides and phosphopeptides were identified by Proteome Discoverer v. 3.1 (Thermo Scientific, Germany). Search parameters included up to two missed cleavages for trypsin digestion, with precursor and fragment mass tolerances set to 10 p.p.m. and 0.02 Da, respectively. Carbamidomethylation (C, +57.021464 Da) and TMT-10plex labeling on N-termini of peptides (+229.162932 Da) were set as static modifications, while oxidation (M, +15.9949 Da), N-termini acetylation (+42.010565 Da), and TMT-10plex (K, +229.162932 Da) were set as dynamic modifications. The phosphorylation (S/T/Y, +79.966Da) was also added as a variable modification for phosphoproteomic data. All results were filtered with a 1 % false discovery rate (FDR).

For quantification of peptides and phosphopeptides, raw intensities of TMT reporter ions were normalized within each TMT set to account for differences in sample loading and systematic variations across channels. Normalization was performed by dividing each TMT channel by the total sum of intensities across all channels within a set.

### 4.14 Intact glycopeptide identification and quantification

Intact glycopeptides were identified using the StrucGP software, which enables de novo glycan structure identification [23]. Briefly, the intact glycopeptide analyses were performed using the built-in glycan branch structure database (containing 17 branch structures) from StrucGP and the *Mus musculus* UniProt protein database, applying the same search parameters as used for proteomic data. Peptides with potential glycosites were screened using the N-X-S/T motif (where X is any amino acid except proline). Additional criteria included the presence of at least two oxonium ions among the top 10 fragment ions in MS/MS spectra to qualify spectra for intact glycopeptide analysis. Mass tolerances were set to 10 ppm for precursor ions and 20 ppm for fragment ions. Identification results were filtered with a 1 % false discovery rate (FDR) for both peptide sequences and glycan structures, estimated by the decoy peptide [48,64] and decoy spectra method [23], respectively. Identified glycopeptide spectra were ranked by scores, with the top-scoring peptide/glycan combination selected as the final identification. To further ensure reliability, a probability-based evaluation was applied to each module of the identified glycan structures [23].

For quantification, TMT reporter ion intensities were extracted from the identified MS/MS spectra of intact glycopeptides. The intensities of TMT reporter ions were first normalized using global scaling factors obtained from global proteomic results to ensure consistency across the ten channels. These normalized intensities were used for quantitative analysis of identified glycopeptide. Median intensities of all PSMs were calculated for each sample and were used to calculate the ratios of intact glycopeptide between two age samples. To evaluate differences of glycopeptide abundances between two age groups, normalized data were statistically compared using two-tailed unpaired t-tests. Glycopeptides with a fold change greater than 1.5 or less than 0.67 and a *p*-value <0.05 were considered differentially expressed.

### 4.15 Bioinformatic analysis

Principal Component Analysis (PCA), bubble plot, volcano plot and sankey diagram were performed using OmicStudio tools at www.omicstudio.cn/tool. Gene ontology (GO) enrichment analysis was conducted with the Database for Annotation, Visualization, and Integrated Discovery (DAVID) (david.ncifcrf.gov) to identify enriched biological process (BP), cellular component (CC), and molecular function (MF) [65]. Kyoto Encyclopedia of Genes and Genomes (KEGG) analysis was performed in Cytoscape v.3.8.2 [66]. These pathway enrichment analyses were performed to search for the associated important pathway information and key glycoproteins. GO enrichment results were visualized with GO-Figure (gitlab.com/evogenlab/GO-Figure). Motif was plotted by bioinformatics.com.cn (last accessed on June 20th, 2024), an online platform for data analysis and visualization [67]. Protein-protein interaction (PPI) network analyses were performed using Search Tool for the Retrieval of Interacting Genes/Proteins (STRING: functional protein association networks (string-db.org)) [68], and interactions with a combined score of >0.4 were selected to construct the PPI networks using Cytoscape [69]. Furthermore, the Cyto-Hubba plugin (apps.cytoscape.org/apps/cytohubba) was used to identify the hub proteins using 12 topological algorithms, including the Percolated Component (EPC), Maximal Clique Centrality (MCC), Density of Maximum Neighborhood Component (DMNC), Edge Maximum Neighborhoods Component (MNC), Betweenness, Stress Paths, Bottleneck (BN), Closeness, Radiality, Eccentricity, Clustering Coefficient, and Degrees [70].

### 4.16 Statistical analyses

Data in the bar plots are shown as the mean ± s.e.m. *P* values < 0.05 were considered to be statistically significant. Blank indicates not significant. All experimental data were analyzed using unpaired t-tests to compare differences between groups (GraphPad v.9.0 Software).

## CRediT authorship contribution statement

**Yongqi Wu:** Writing – review & editing, Writing – original draft, Visualization, Validation, Software, Methodology, Investigation, Formal analysis, Data curation, Conceptualization. **Zhida Zhang:** Resources, Data curation. **Yongchao Xu:** Visualization, Formal analysis, Data curation. **Yingjie Zhang:** Visualization. **Lin Chen:** Visualization, Data curation. **Yiwen Zhang:** Data curation. **Ke Hou:** Data curation. **Muyao Yang:** Data curation. **Zhehui Jin:** Data curation. **Yinli Cai:** Software. **Jiayu Zhao:** Software. **Shisheng Sun:** Writing – review & editing, Supervision, Project administration, Funding acquisition.

## Declaration of competing interest

The authors declare that they have no known competing financial interests or personal relationships that could have appeared to influence the work reported in this paper.

## Data Availability

The mass spectrometry proteomics data have been deposited to the ProteomeXchange Consortium (https://proteomecentral.proteomexchange.org) via the iProX partner repositor [71] with the dataset identifier PXD056126.
