# Supplementary material for: A high-resolution N-glycoproteome landscape of aging mouse ovary
Source: Redox Biol. 2025 Mar 7;81:103584. doi: 10.1016/j.redox.2025.103584 (PMC11938160; doi:10.1016/j.redox.2025.103584)
Supplement: Multimedia component 9 [file mmc9.pdf]

Supplementary Information for:

A High-resolution *N*-Glycoproteome Landscape of Aging Mouse Ovary

Yongqi Wu, Zhida Zhang, Yongchao Xu, Yingjie Zhang, Lin Chen, Yiwen Zhang, Ke Hou, Muyao Yang, Zhehui Jin, Yingli Cai, Jiayu Zhao, Shisheng Sun*.

Laboratory for Disease Glycoproteomics, College of Life Sciences, Northwest University, Xi'an, 710069, P. R. China

*Correspondence: suns@nwu.edu.cn

Supplementary information

1. Supplementary Figures

Figure S1 Evaluation of the mouse model for ovarian aging studies and the quality of mass spectrometry data.

Figure S2 Characterization of glycoproteomic data from mouse ovaries.

Figure S3 Identification of glycans on mouse ovarian glycoproteins with different antenna numbers.

Figure S4 Representative MS/MS Spectra of three glycan subtypes, four core structures, and seventeen branch structures (A separate PDF file). Related to Figure. 1H-1J.

Figure S5 The percentages of each glycan sub-structure on glycoproteins that were associated with different biological processes and molecular functions.

Figure S6 Site-specific glycan mapping of ZP1, ZP2 and ZP3 in mouse ovaries.

2. Supplementary Tables (separate excel files)

Table S1 Summary of global proteins, glycoproteins, and phosphoproteins identified in mouse ovaries.

Table S2 Intact glycopeptides identified in mouse ovaries with FDR<1% applied at the glycosite-containing peptide level or at both glycosite-containing peptide and glycan levels.

Table S3 List of ovarian glycoproteins enriched in different subcellular components.

Table S4 Quantitative glycopeptides between young and middle-aged mouse ovaries based on the TMT-labeled quantification method.

Table S5 LacdiNAc-containing glycopeptides identified from mouse ovaries and site-specific glycans identified from zona pellucida glycoproteins.

Table S6 Quantification of differential sialoglycopeptides with four different types of

branch structures in aged ovaries.

Table S7 Quantitative phosphopeptides and proteins between young and middle-aged mouse ovaries based on the TMT-labeled quantification method.

Table S8 Mouse ovarian glycopeptides associated with ferroptosis or apoptosis.

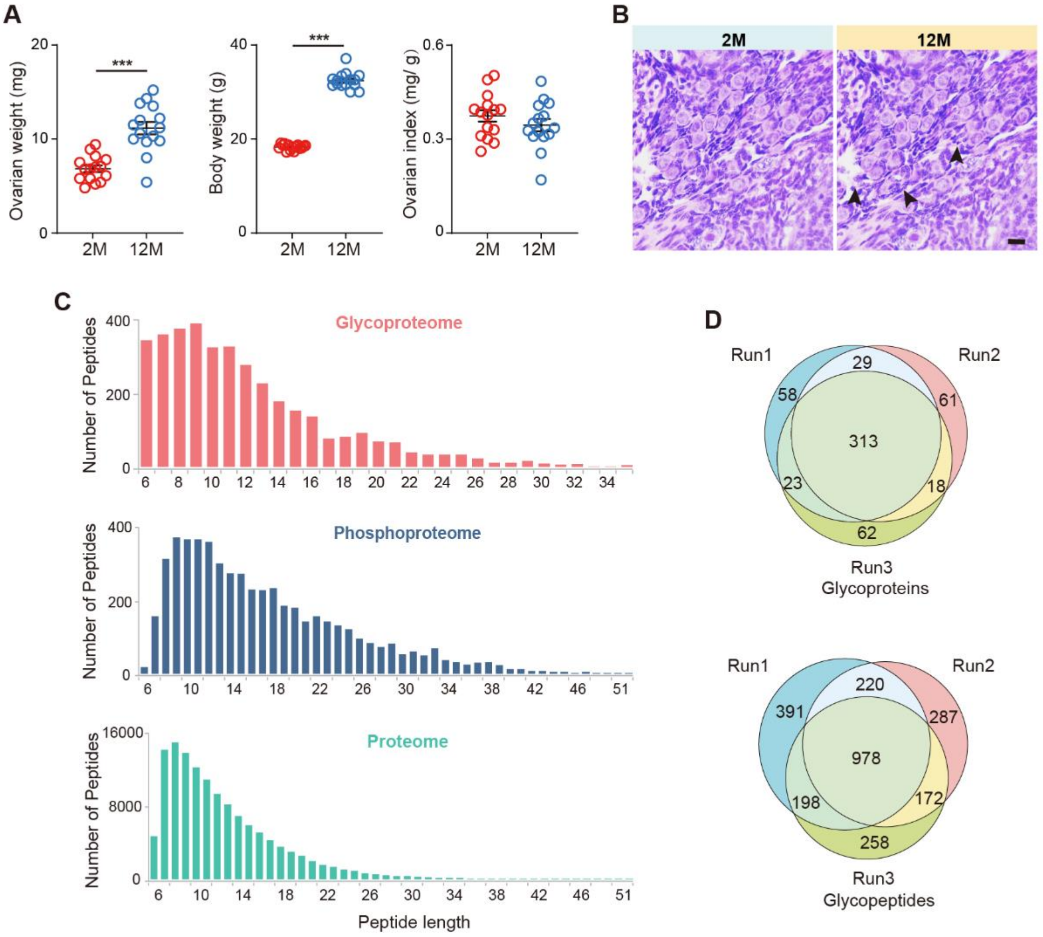

Figure S1. Evaluation of the mouse model for ovarian aging studies and the quality of mass spectrometry data. (A) Ovarian weights, body weights and ovarian indexes in two age groups of mice. (B) H&E staining to show the primordial follicles from mouse ovaries with 2-month and 12-month-old. Black arrowheads indicate the rare primordial follicles in aged ovaries. Scale bar, 20 µm. (C) Distributions of peptide length (amino acid numbers) identified from intact glycopeptides (glycoproteome), phosphopeptides (phosphoproteome), and all peptides (proteome). (D) Evaluation of LC-MS reproducibility based on the overlaps of glycoproteins and glycopeptides identified from triplicate LC-MS/MS analyses of a single fraction. Data presented as mean ± s.e.m. *P< 0.05, **P< 0.01, ***P < 0.005 by two-tailed t-test (A). 2M, 2-month-old. 12M, 12-month-old.

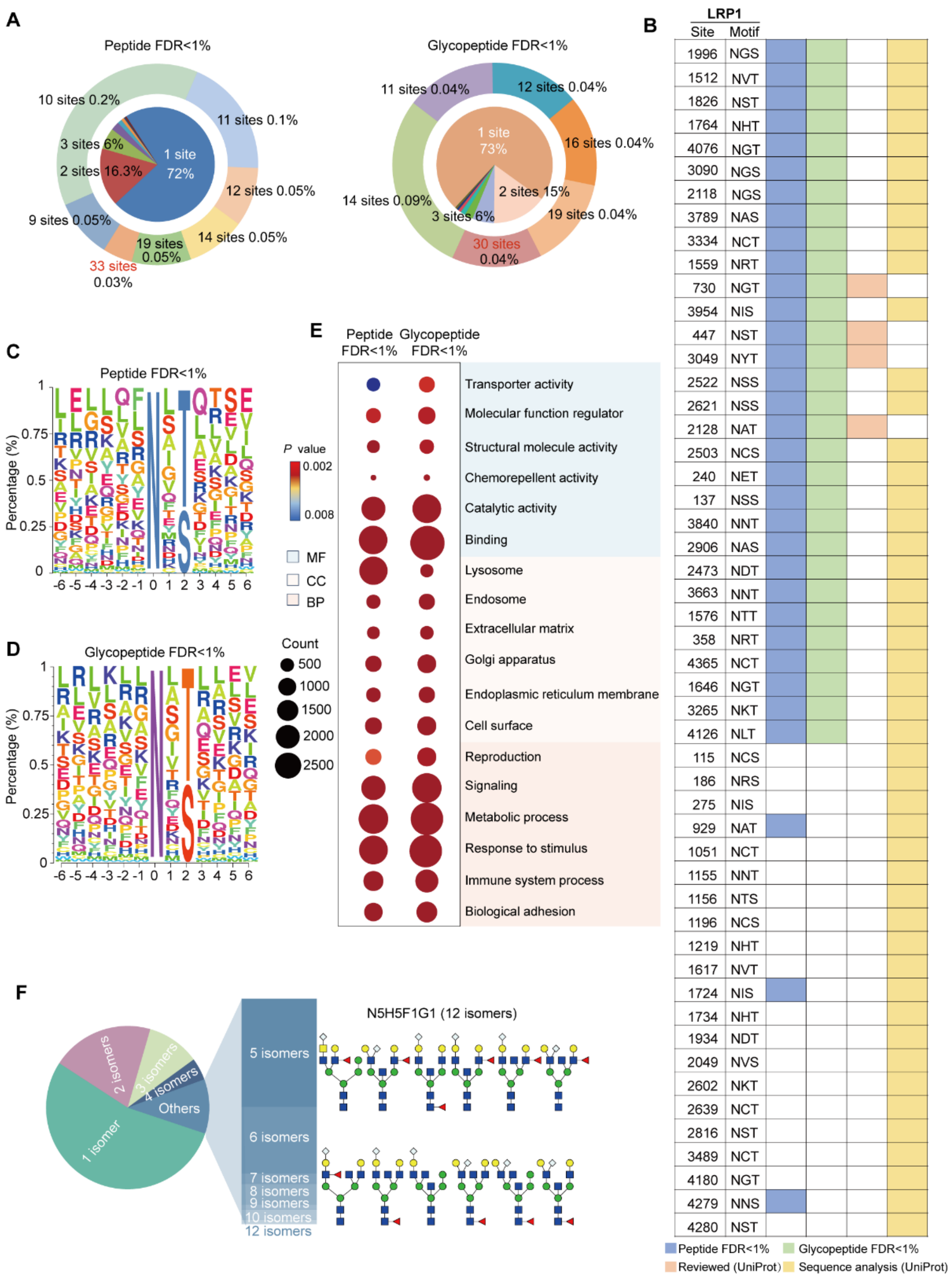

Figure S2. Characterization of glycoproteomic data from mouse ovaries. (A) Number of N-linked glycosylation sites identified on individual glycoproteins. The identified glycoproteins and glycosites were controlled by the FDR < 1% either at the glycosite-containing peptide level (left) or at both glycosite-containing peptide and glycan levels (right). (B) Comparison of *N*-glycosites identified from Prolow-density lipoprotein receptor-related protein 1 (LRP1) with predicted N-glycosites in UniProt database. The LRP1 contains the highest number of identified glycosites. (C and D) Motif analysis of glycosites identified based on FDR<1% at the peptide level (C) or at

both peptide and glycan levels (D). (E) Gene Ontology enrichment of glycoproteins identified from mouse ovaries. (F) Glycan isomers identified in mouse ovaries. Up to 12 glycan isomers were identified from the glycan composition of N5H5F1G1. N: HexNAc; H: hexose; F: fucose; G Neu5Gc.

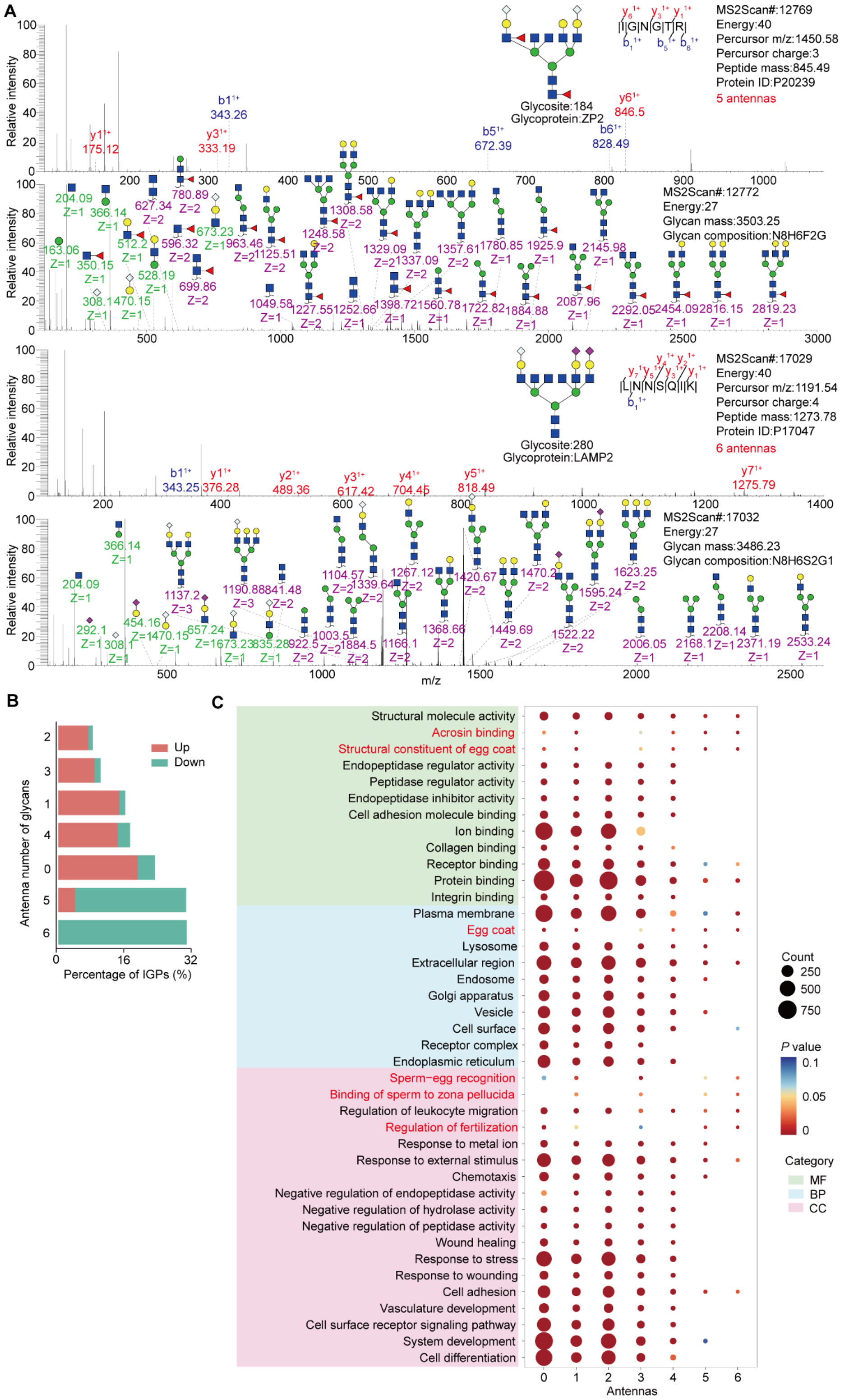

a

Figure S3. Identification of glycans on mouse ovarian glycoproteins with different antenna numbers. (A and B) Representative spectra of two glycopeptides with 5-6 antennas. (C) Distribution of differential glycopeptides containing different number of antennas. DEGPs, differentially expressed glycopeptides. (D) Representative Gene Ontology terms for glycopeptides with different antenna numbers.

A separate PDF file

Figure S4 Representative MS/MS Spectra of three glycan subtypes, four core structures, and seventeen branch structures (.pdf). Related to Figure. 1H-1J.

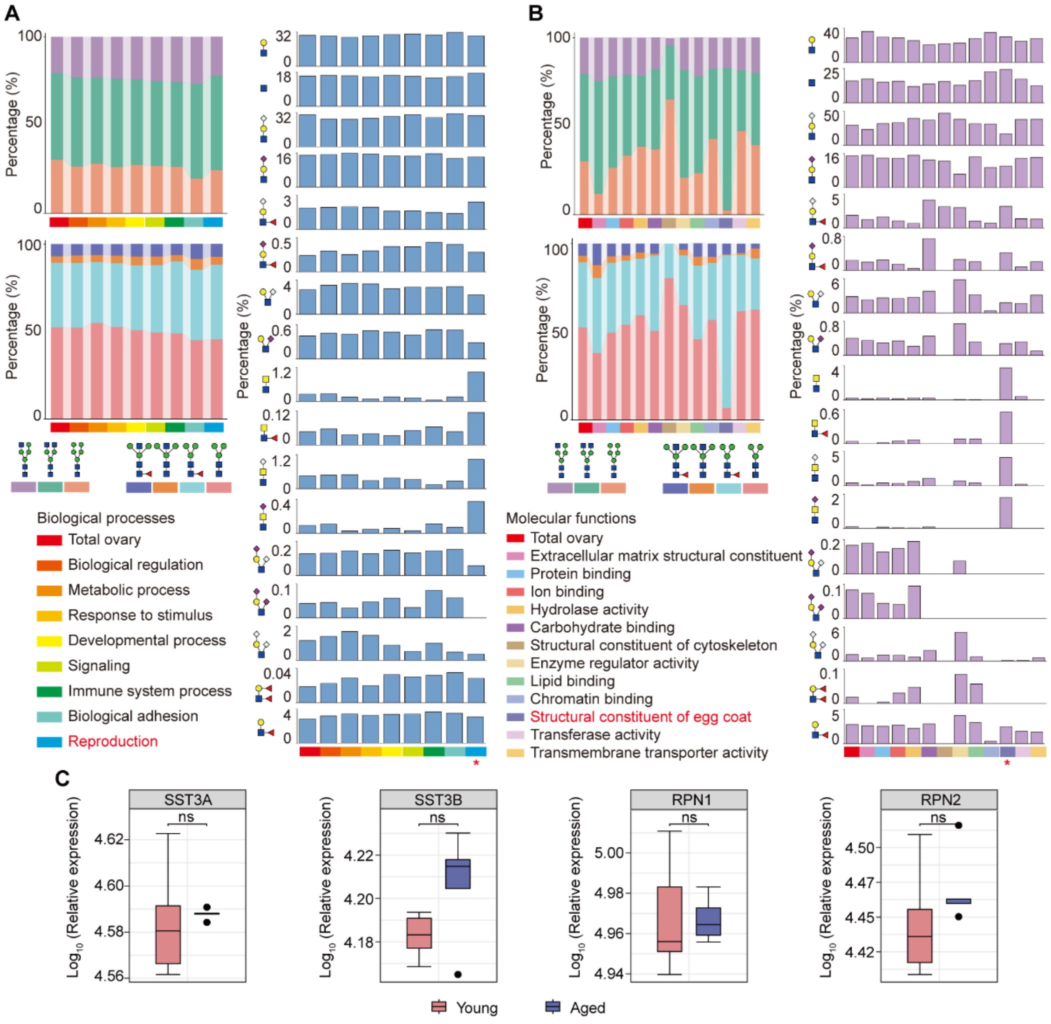

Figure S5. The percentages of each glycan sub-structure on glycoproteins that were associated with different biological processes and molecular functions. (A and B) Percentages of unique glycopeptides with glycan subtypes, core and branch structures among different biological processes (A) and molecular functions (B). (C) Relative protein expression of oligosaccharyltransferase (OST) subunits.

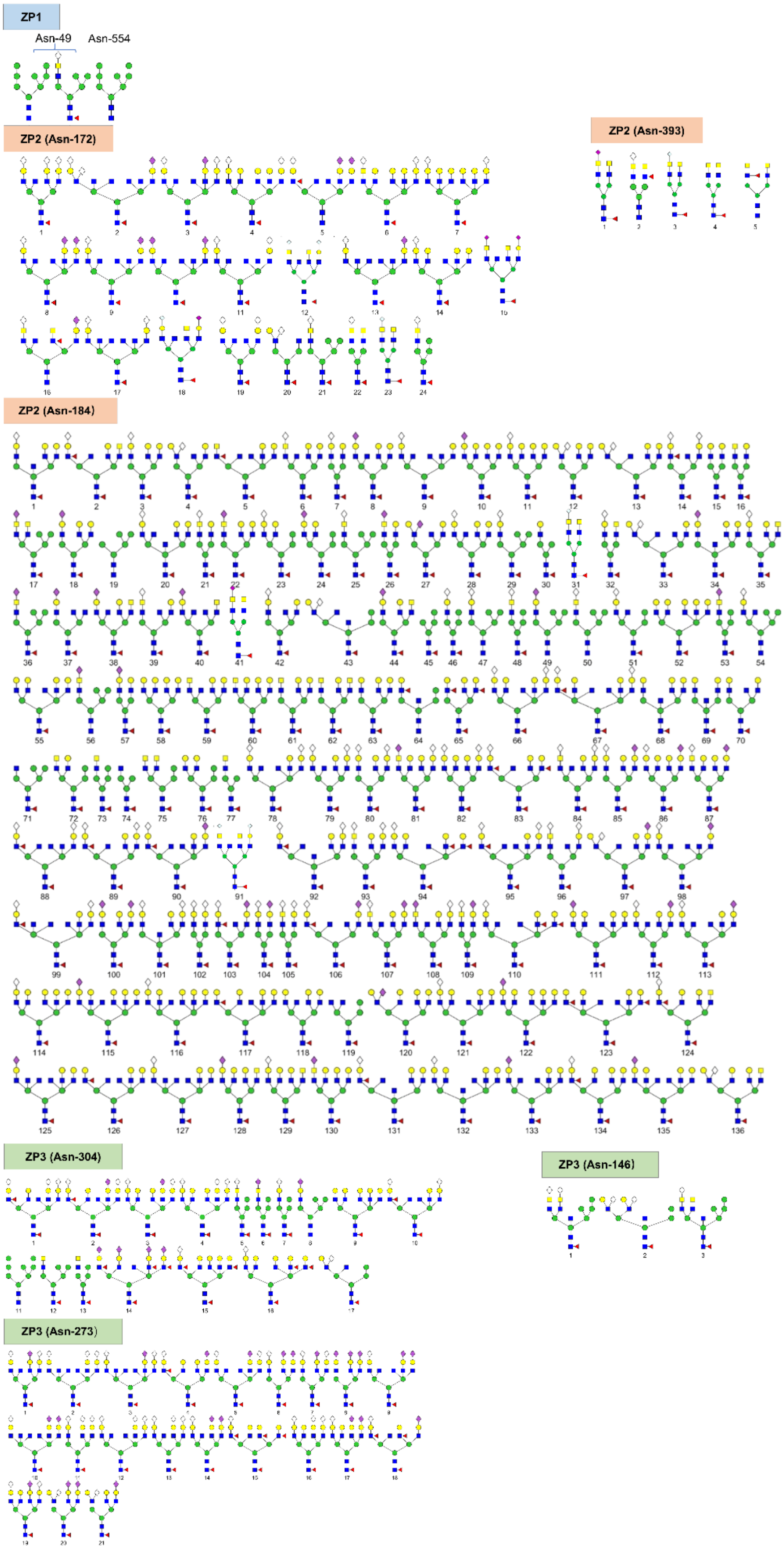

Figure S6. Site-specific glycan mapping of ZP1, ZP2 and ZP3 in mouse ovaries.
