# Supplementary material for: A high-resolution N-glycoproteome landscape of aging mouse ovary
Source: Redox Biol. 2025 Mar 7;81:103584. doi: 10.1016/j.redox.2025.103584 (PMC11938160; doi:10.1016/j.redox.2025.103584)
Supplement: Multimedia component 10 [file mmc10.pdf]

Figure S4

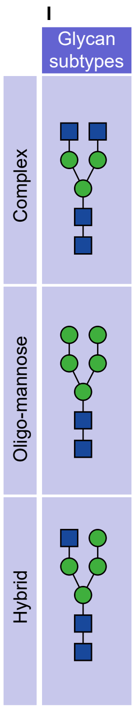

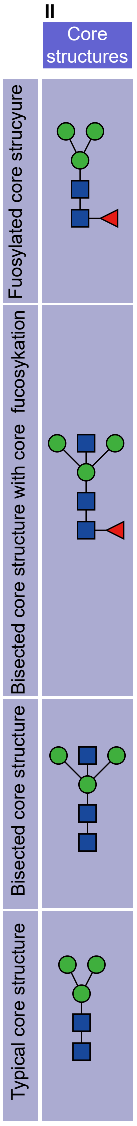

III

NO.	Codes	Branch structures	Fragment Ions	Page
1	E2e		204.1	4
2	E2F1fe		366.1, 204.1	4
3	E2F1fF3fe		675.2, 495.2, 292.1	5
4	E2F1fF4fe		673.2, 511.2, 308.1	5
5	E2F1fF5fe		512.2, 350.1	5
6	E2F1G3gfe		657.2, 454.2, 292.1	6
7	E2F1G3gfF3fe		948.3, 657.2, 675.2, 495.2	6
8	E2F1G3gfF4fe		965.3, 657.2, 673.2, 511.2, 308.1, 292.1	6
9	E2F1G3gfF5fe		803.3, 657.2, 512.2, 350.1, 292.1	7
10	E2F1G4gfe		673.2, 470.2, 308.1	7
11	E2F1G4gfF4fe		980.3, 673.2, 673.2, 511.2, 308.1	7
12	E2F1G4gfF5fe		819.3, 673.2, 512.2, 350.1, 308.1	8
13	E2F1G5gfF5fe		658.3, 512.2, 350.1, 308.1	8
14	E2F2fe		407.2	8
15	E2F2fF5fe		553.2, 407.2, 350.1	9
16	E2F2G3gfe		698.3, 495.2, 407.2, 292.1	9
17	E2F2G4gfe		714.3, 511.2, 407.2, 308.1	9

I. Glycan subtypes (3)

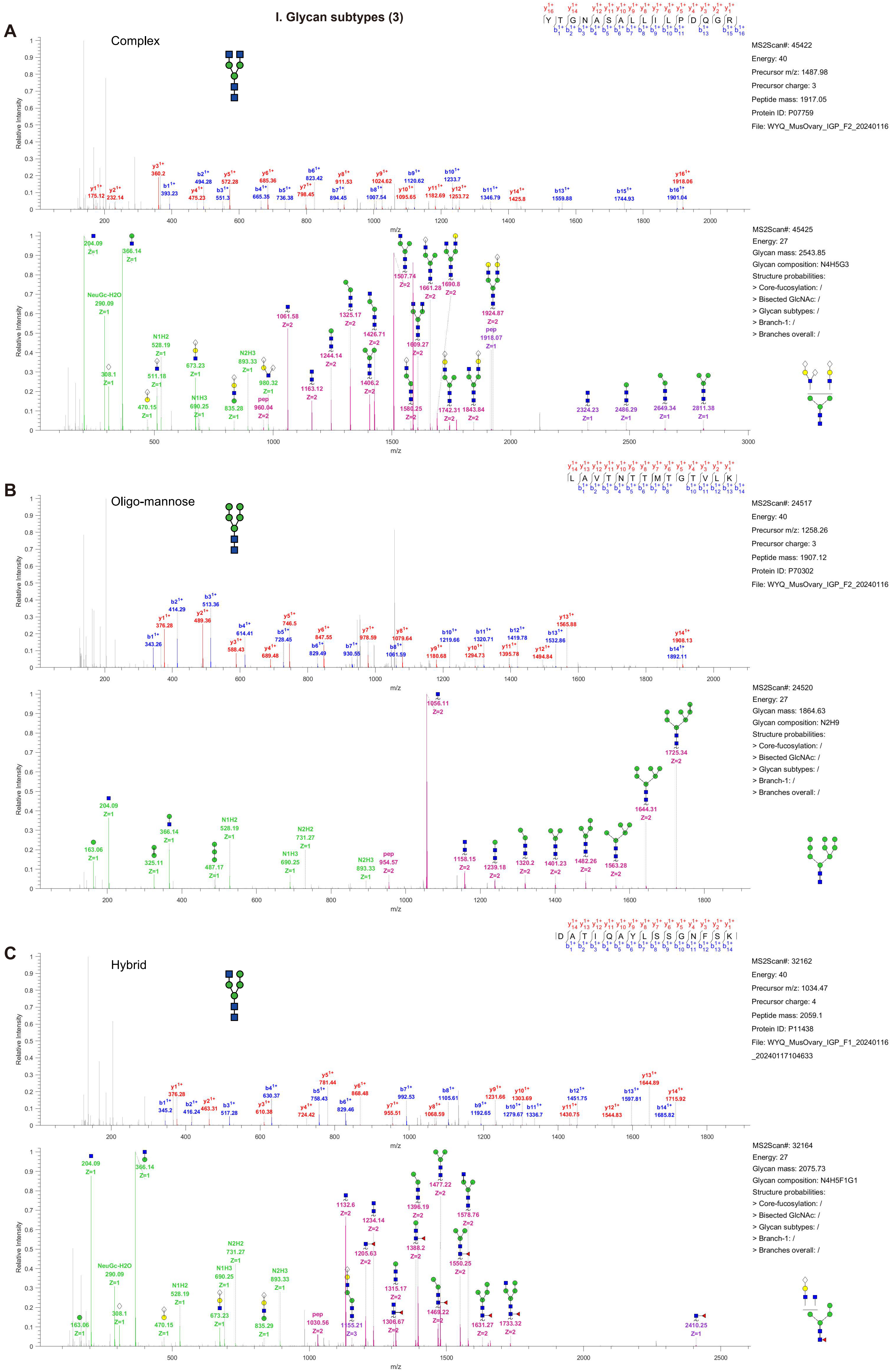

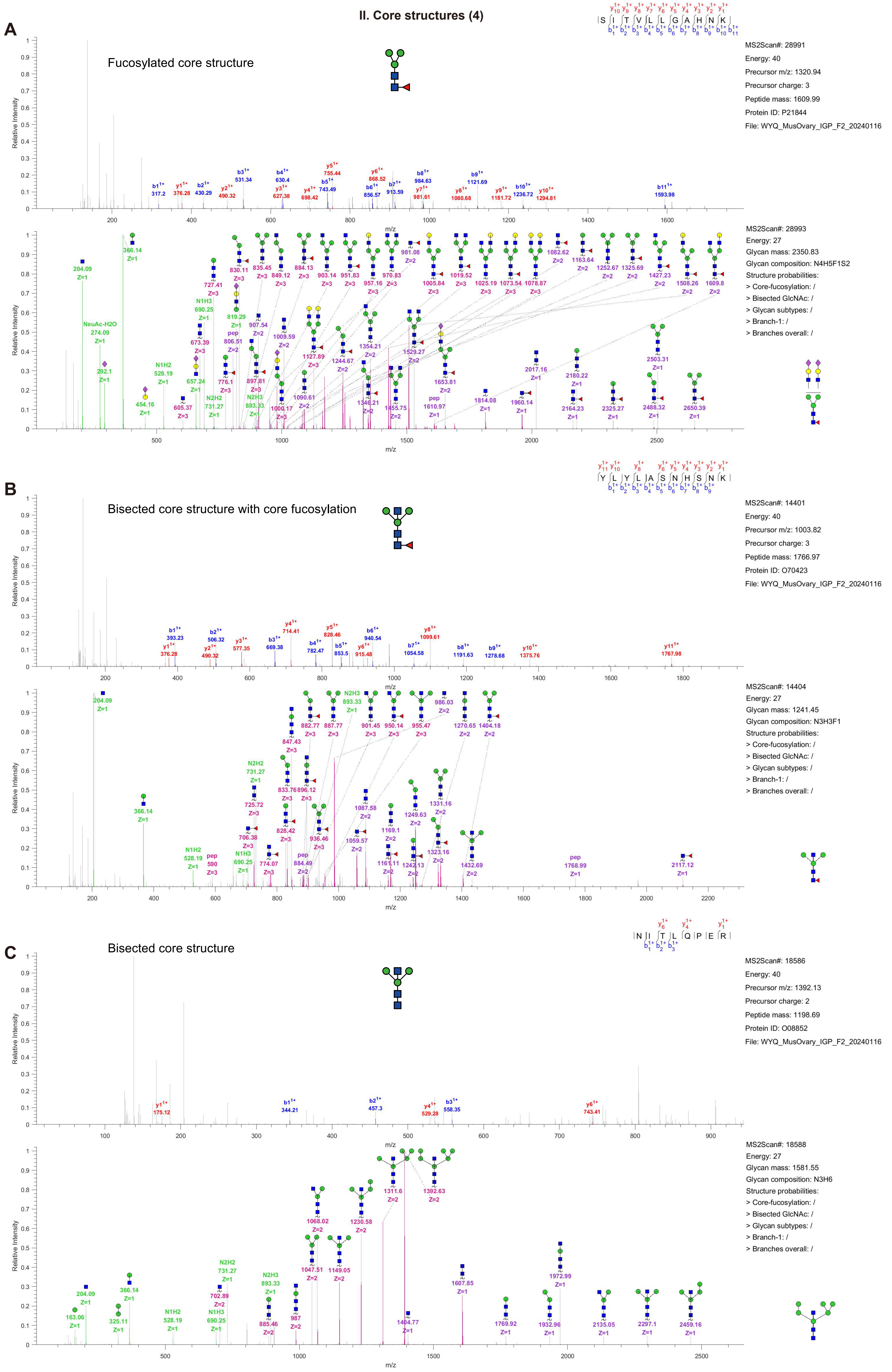
II. Core structures (4)
A
Fucosylated core structure
S I T V L L G A H N K
MS2Scan#: 28991
Energy: 40
Precursor m/z: 1320.94
Precursor charge: 3
Peptide mass: 1609.99
Protein ID: P21844
File: WYQ_MusOvary_IGP_F2_20240116
Relative Intensity
m/z
MS2Scan#: 28993
Energy: 27
Glycan mass: 2350.83
Glycan composition: N4H5F1S2
Structure probabilities:
> Core-fucosylation: /
> Bisected GlcNAc: /
> Glycan subtypes: /
> Branch-1: /
> Branches overall: /
B
Bisected core structure with core fucosylation
Y L Y L A S N H S N K
MS2Scan#: 14401
Energy: 40
Precursor m/z: 1003.82
Precursor charge: 3
Peptide mass: 1766.97
Protein ID: O70423
File: WYQ_MusOvary_IGP_F2_20240116
MS2Scan#: 14404
Energy: 27
Glycan mass: 1241.45
Glycan composition: N3H3F1
Structure probabilities:
> Core-fucosylation: /
> Bisected GlcNAc: /
> Glycan subtypes: /
> Branch-1: /
> Branches overall: /
C
Bisected core structure
N I T L Q P E R
MS2Scan#: 18586
Energy: 40
Precursor m/z: 1392.13
Precursor charge: 2
Peptide mass: 1198.69
Protein ID: O08852
File: WYQ_MusOvary_IGP_F2_20240116
MS2Scan#: 18588
Energy: 27
Glycan mass: 1581.55
Glycan composition: N3H6
Structure probabilities:
> Core-fucosylation: /
> Bisected GlcNAc: /
> Glycan subtypes: /
> Branch-1: /
> Branches overall: /

D

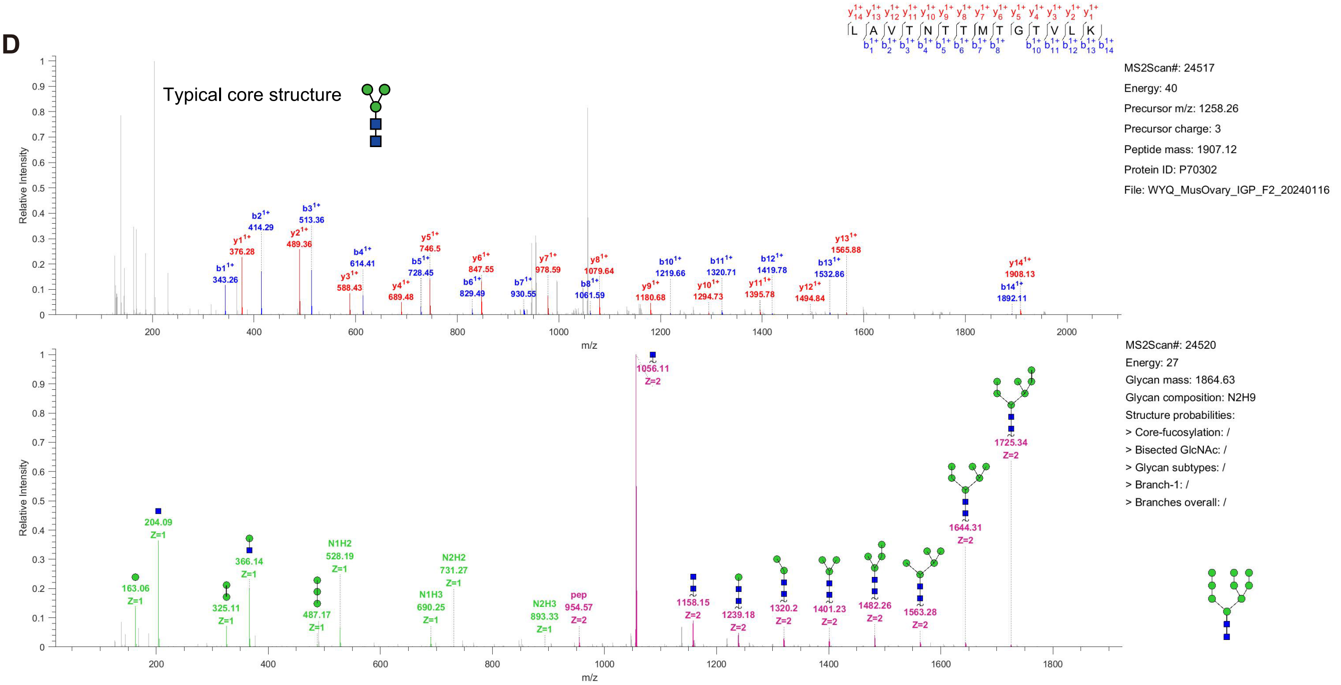

III. Branch structures (17)

A

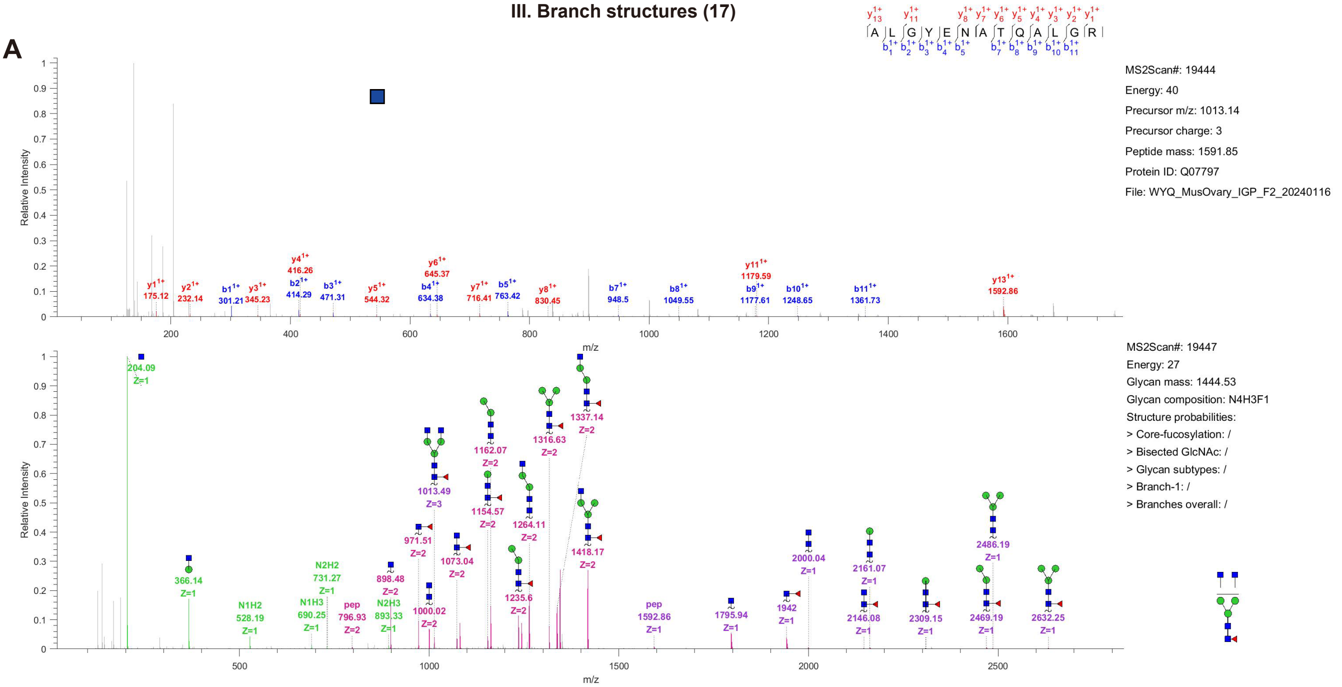

B

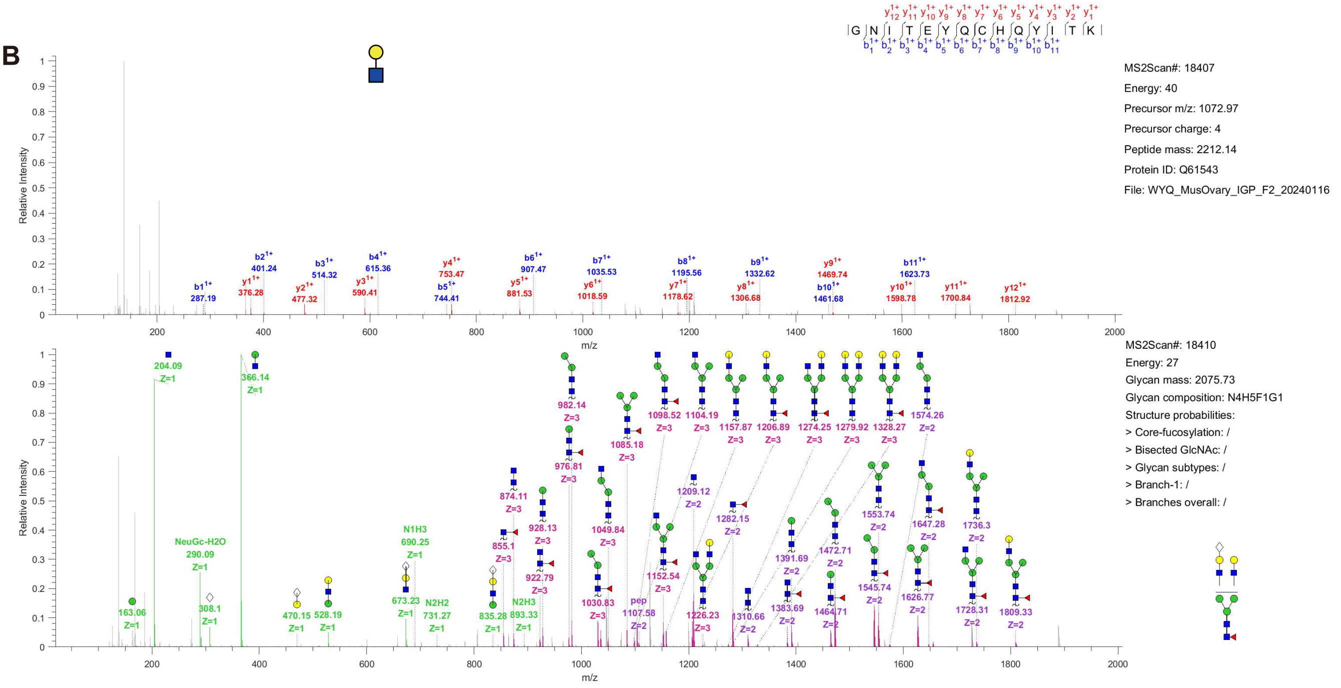

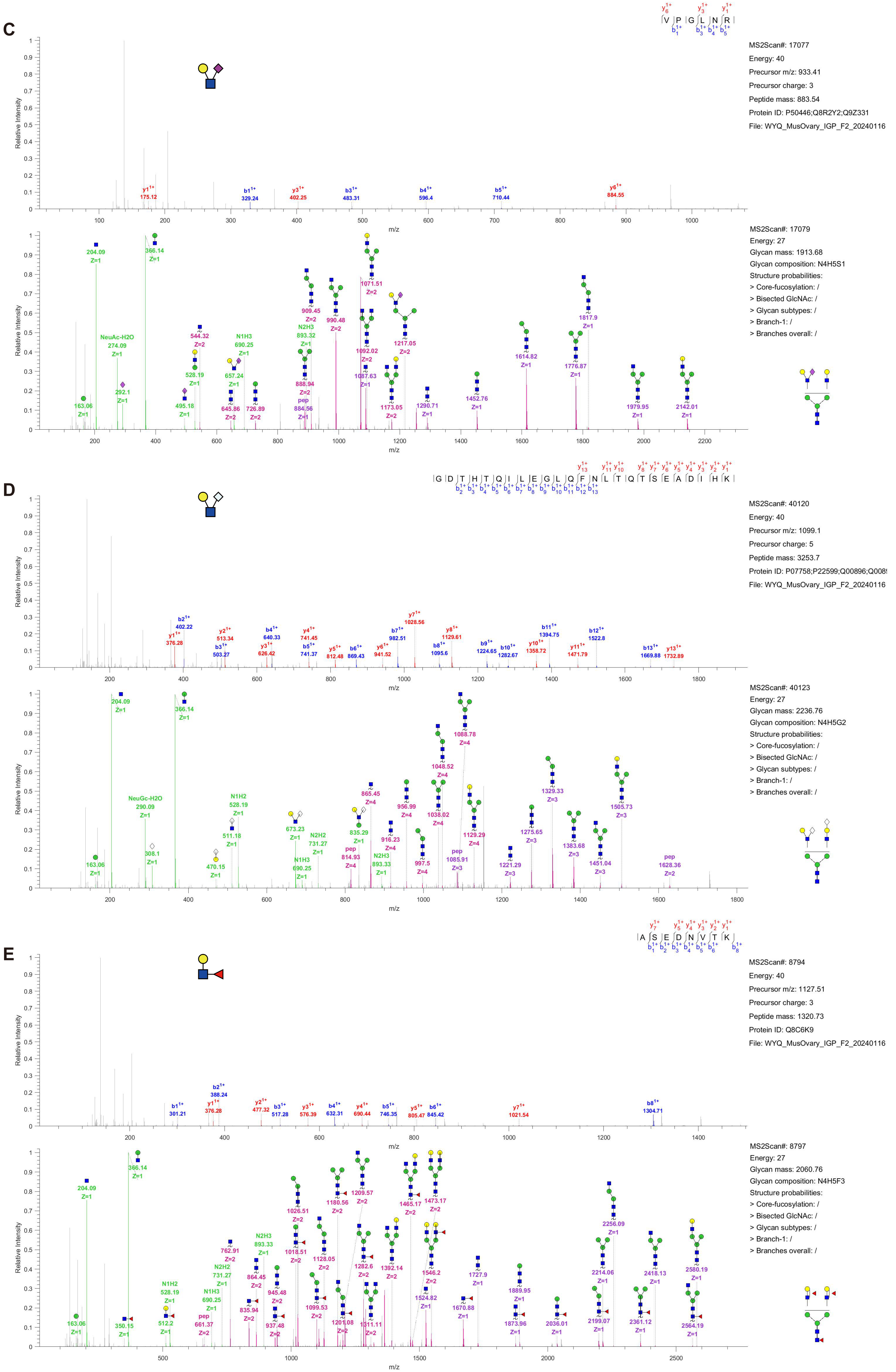

C
V P G L N R
MS2Scan#: 17077
Energy: 40
Precursor m/z: 933.41
Precursor charge: 3
Peptide mass: 883.54
Protein ID: P50446;Q8R2Y2;Q9Z331
File: WYQ_MusOvary_IGP_F2_20240116
MS2Scan#: 17079
Energy: 27
Glycan mass: 1913.68
Glycan composition: N4H5S1
Structure probabilities:
> Core-fucosylation: /
> Bisected GlcNAc: /
> Glycan subtypes: /
> Branch-1: /
> Branches overall: /
D
G D T H T Q I L E G L Q F N L T Q T S E A D I H K
MS2Scan#: 40120
Energy: 40
Precursor m/z: 1099.1
Precursor charge: 5
Peptide mass: 3253.7
Protein ID: P07758;P22599;Q00896;Q008
File: WYQ_MusOvary_IGP_F2_20240116
MS2Scan#: 40123
Energy: 27
Glycan mass: 2236.76
Glycan composition: N4H5G2
Structure probabilities:
> Core-fucosylation: /
> Bisected GlcNAc: /
> Glycan subtypes: /
> Branch-1: /
> Branches overall: /
E
A S E D N V T K
MS2Scan#: 8794
Energy: 40
Precursor m/z: 1127.51
Precursor charge: 3
Peptide mass: 1320.73
Protein ID: Q8C6K9
File: WYQ_MusOvary_IGP_F2_20240116
MS2Scan#: 8797
Energy: 27
Glycan mass: 2060.76
Glycan composition: N4H5F3
Structure probabilities:
> Core-fucosylation: /
> Bisected GlcNAc: /
> Glycan subtypes: /
> Branch-1: /
> Branches overall: /
Relative Intensity
m/z

F

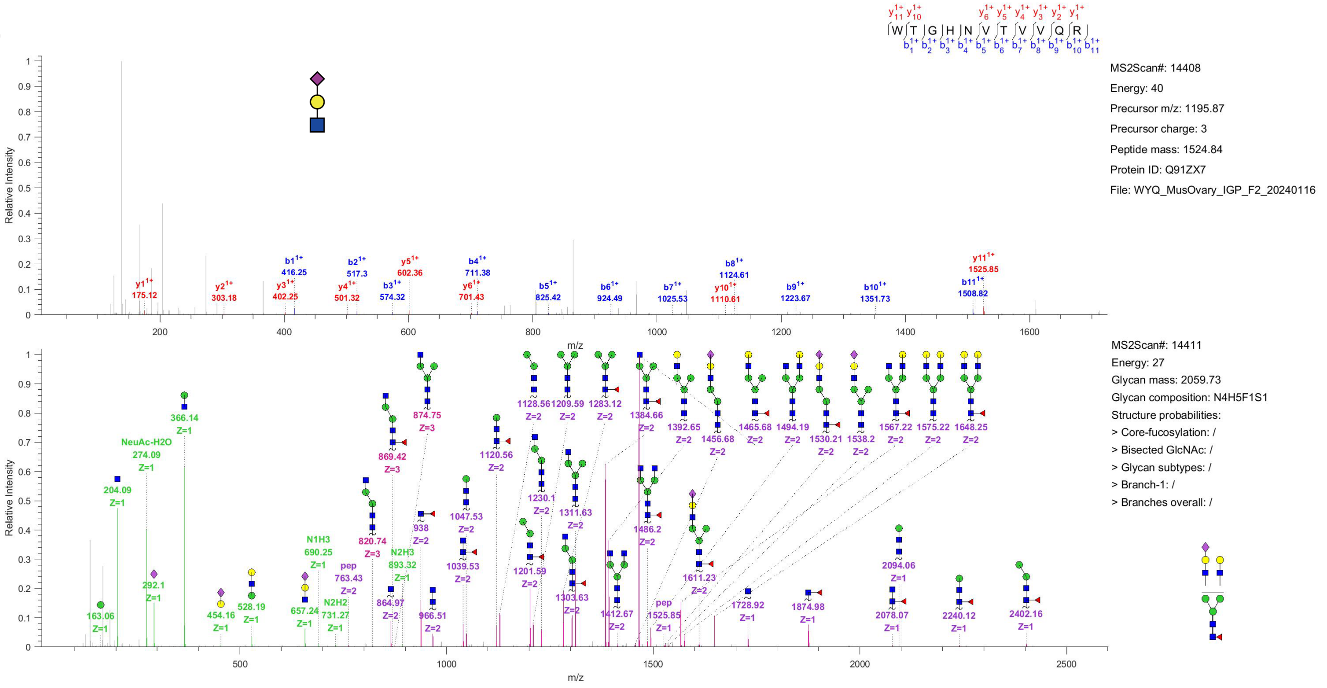
W T G H N V T V V Q R
MS2Scan#: 14408
Energy: 40
Precursor m/z: 1195.87
Precursor charge: 3
Peptide mass: 1524.84
Protein ID: Q91ZX7
File: WYQ_MusOvary_IGP_F2_20240116
MS2Scan#: 14411
Energy: 27
Glycan mass: 2059.73
Glycan composition: N4H5F1S1
Structure probabilities:
> Core-fucosylation: /
> Bisected GlcNAc: /
> Glycan subtypes: /
> Branch-1: /
> Branches overall: /
Relative Intensity
m/z

G

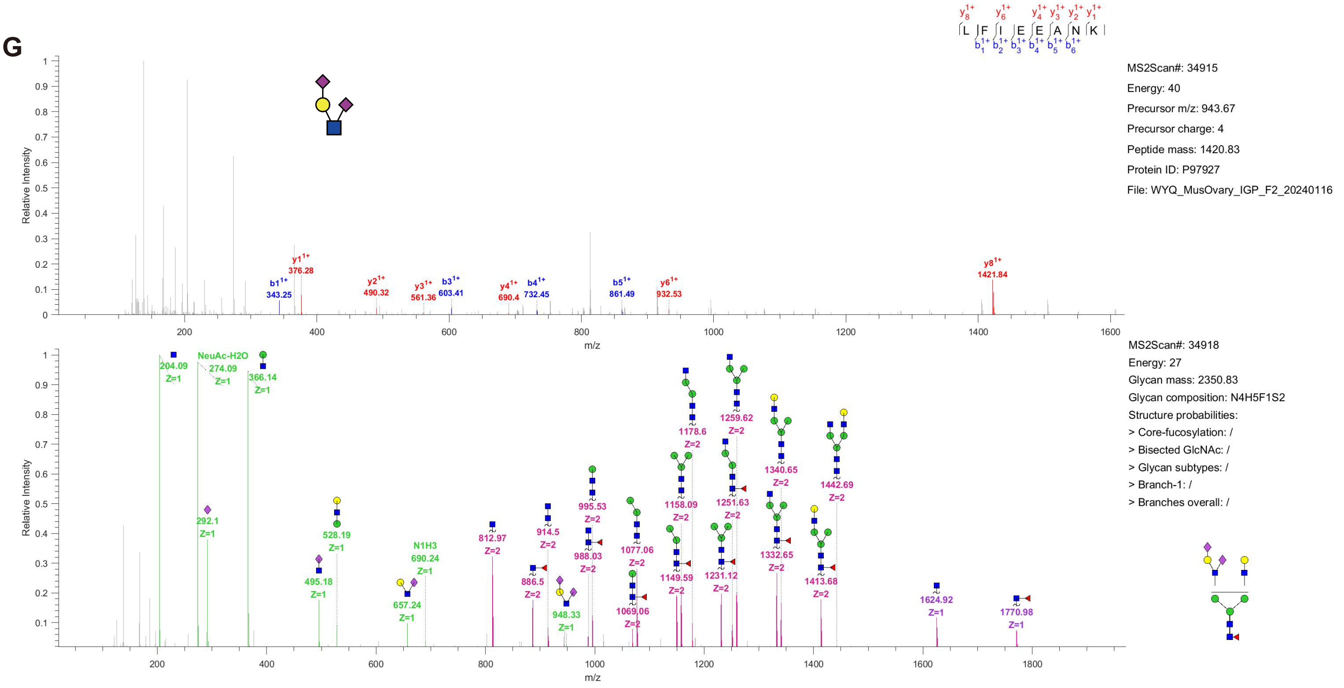
L F I E E A N K
MS2Scan#: 34915
Energy: 40
Precursor m/z: 943.67
Precursor charge: 4
Peptide mass: 1420.83
Protein ID: P97927
File: WYQ_MusOvary_IGP_F2_20240116
MS2Scan#: 34918
Energy: 27
Glycan mass: 2350.83
Glycan composition: N4H5F1S2
Structure probabilities:
> Core-fucosylation: /
> Bisected GlcNAc: /
> Glycan subtypes: /
> Branch-1: /
> Branches overall: /
Relative Intensity
m/z

H

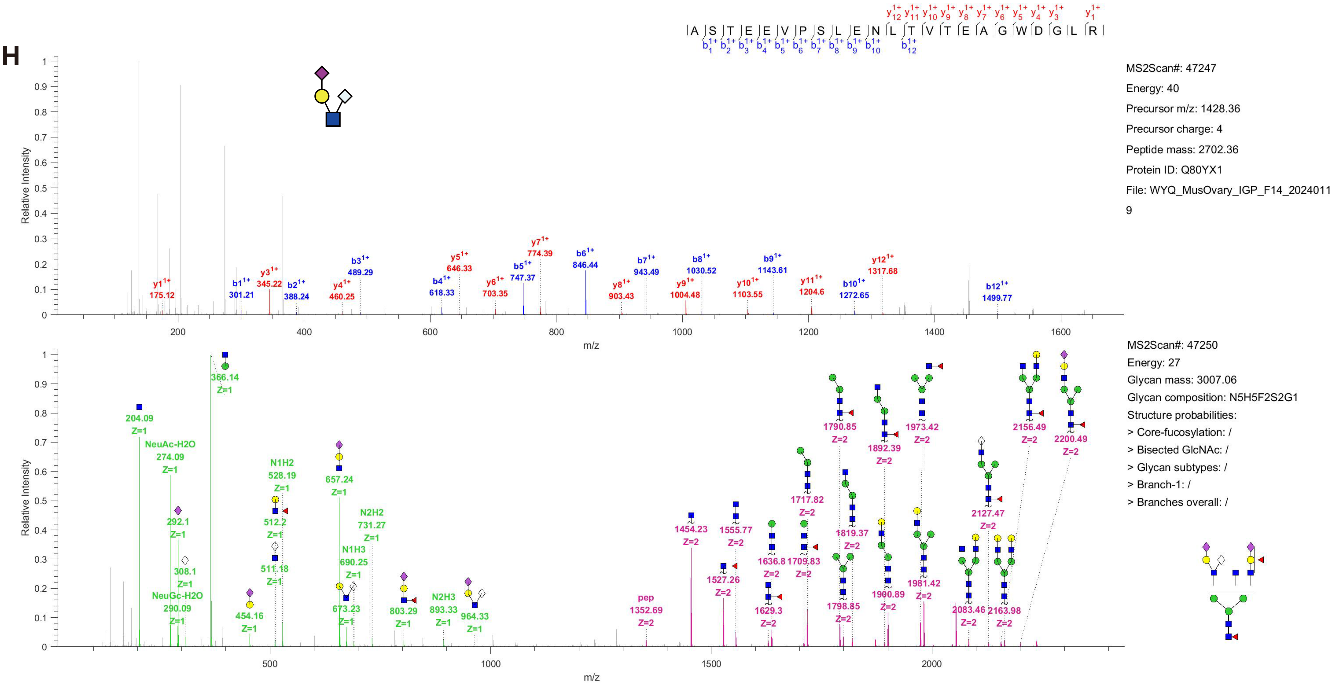
A S T E E V P S L E N L T V T E A G W D G L R
MS2Scan#: 47247
Energy: 40
Precursor m/z: 1428.36
Precursor charge: 4
Peptide mass: 2702.36
Protein ID: Q80YX1
File: WYQ_MusOvary_IGP_F14_2024011
9
MS2Scan#: 47250
Energy: 27
Glycan mass: 3007.06
Glycan composition: N5H5F2S2G1
Structure probabilities:
> Core-fucosylation: /
> Bisected GlcNAc: /
> Glycan subtypes: /
> Branch-1: /
> Branches overall: /
Relative Intensity
m/z

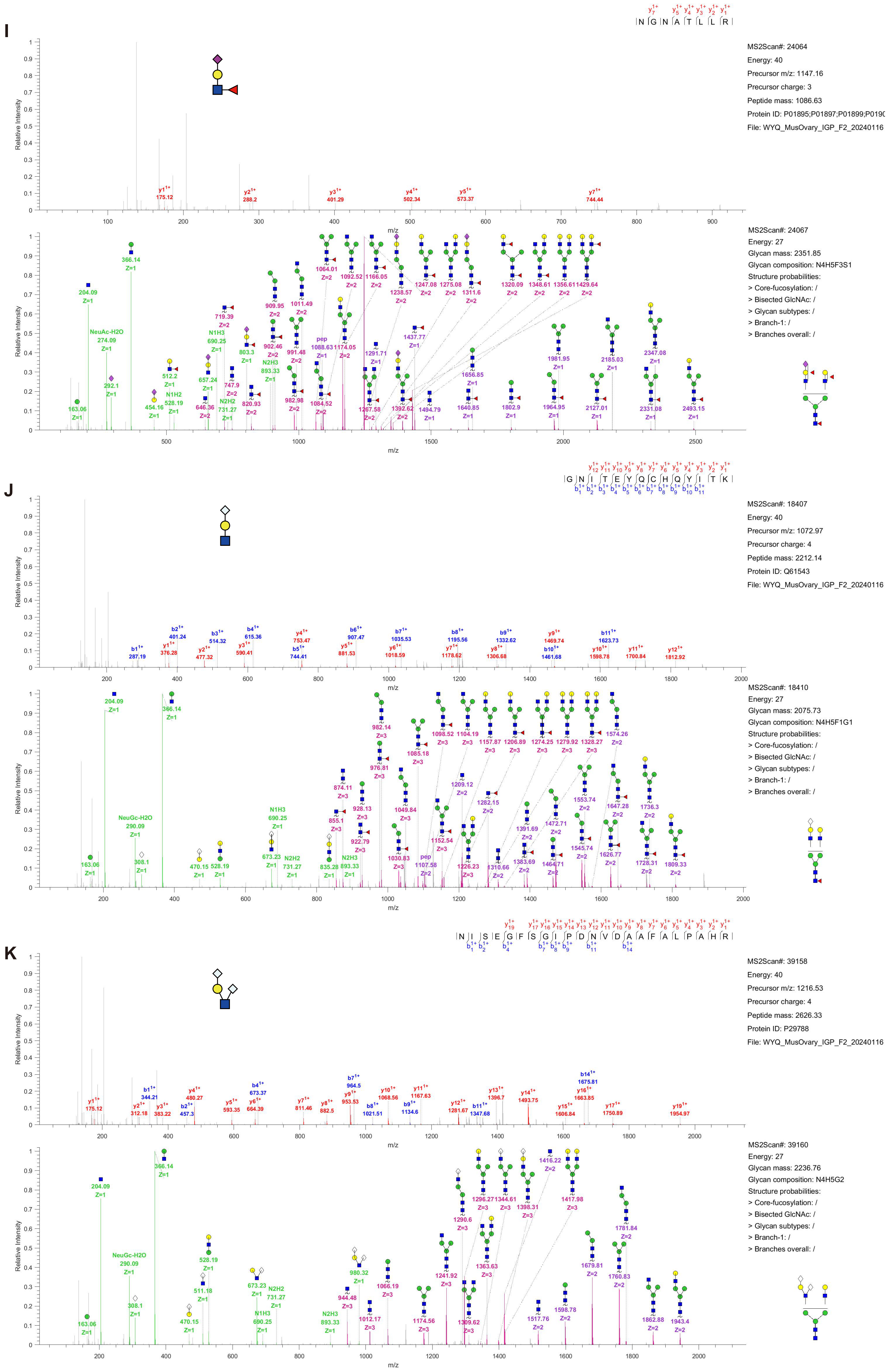

I
N G N A T L L R
MS2Scan#: 24064
Energy: 40
Precursor m/z: 1147.16
Precursor charge: 3
Peptide mass: 1086.63
Protein ID: P01895;P01897;P01899;P019C
File: WYQ_MusOvary_IGP_F2_20240116
MS2Scan#: 24067
Energy: 27
Glycan mass: 2351.85
Glycan composition: N4H5F3S1
Structure probabilities:
> Core-fucosylation: /
> Bisected GlcNAc: /
> Glycan subtypes: /
> Branch-1: /
> Branches overall: /
Relative Intensity
m/z
J
G N I T E Y Q C H Q Y I T K
MS2Scan#: 18407
Energy: 40
Precursor m/z: 1072.97
Precursor charge: 4
Peptide mass: 2212.14
Protein ID: Q61543
File: WYQ_MusOvary_IGP_F2_20240116
MS2Scan#: 18410
Energy: 27
Glycan mass: 2075.73
Glycan composition: N4H5F1G1
Structure probabilities:
> Core-fucosylation: /
> Bisected GlcNAc: /
> Glycan subtypes: /
> Branch-1: /
> Branches overall: /
K
N I S E G F S G I P D N V D A A F A L P A H R
MS2Scan#: 39158
Energy: 40
Precursor m/z: 1216.53
Precursor charge: 4
Peptide mass: 2626.33
Protein ID: P29788
File: WYQ_MusOvary_IGP_F2_20240116
MS2Scan#: 39160
Energy: 27
Glycan mass: 2236.76
Glycan composition: N4H5G2
Structure probabilities:
> Core-fucosylation: /
> Bisected GlcNAc: /
> Glycan subtypes: /
> Branch-1: /
> Branches overall: /

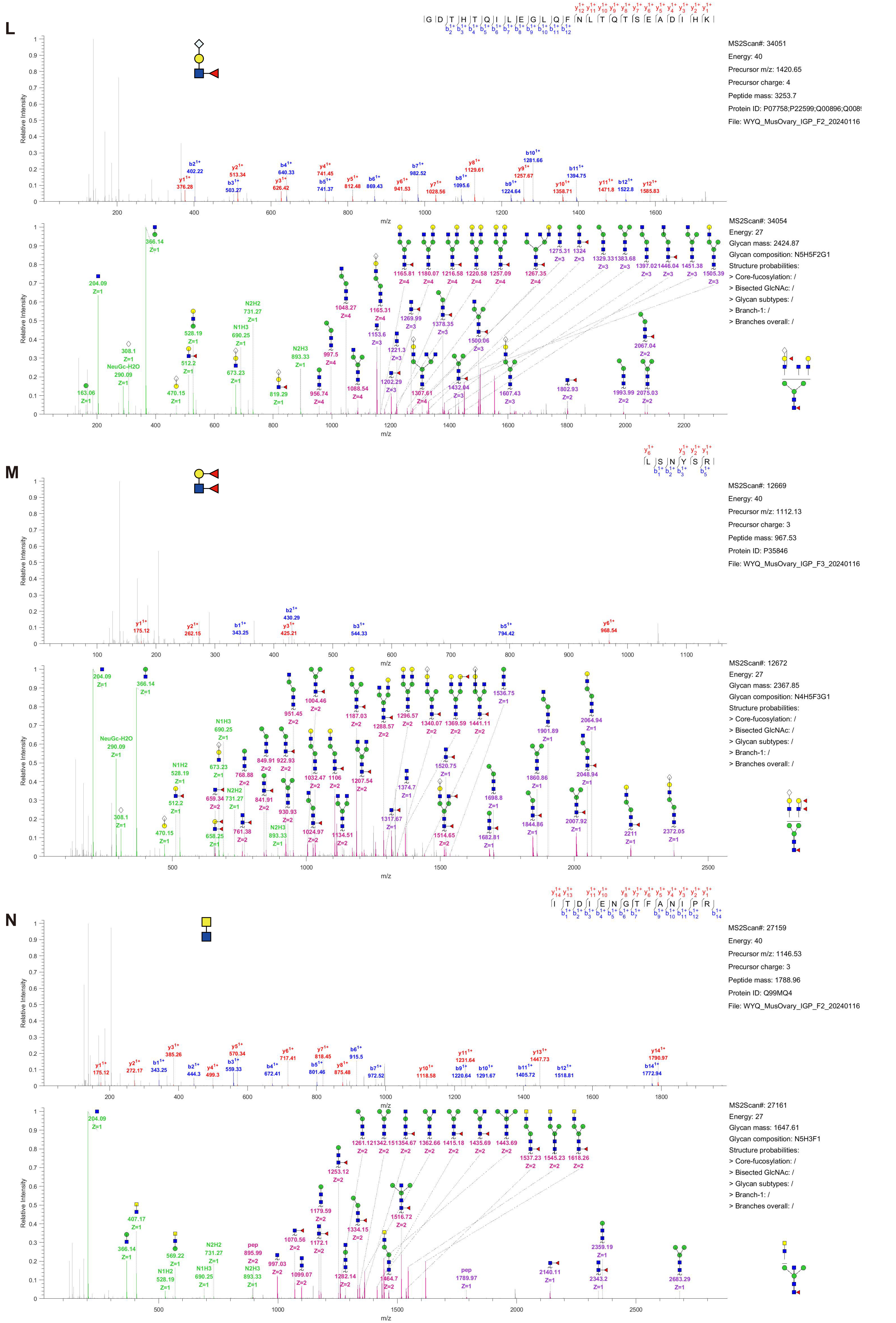
L
G D T H T Q I L E G L Q F N L T Q T S E A D I H K
MS2Scan#: 34051
Energy: 40
Precursor m/z: 1420.65
Precursor charge: 4
Peptide mass: 3253.7
Protein ID: P07758;P22599;Q00896;Q008
File: WYQ_MusOvary_IGP_F2_20240116
MS2Scan#: 34054
Energy: 27
Glycan mass: 2424.87
Glycan composition: N5H5F2G1
Structure probabilities:
> Core-fucosylation: /
> Bisected GlcNAc: /
> Glycan subtypes: /
> Branch-1: /
> Branches overall: /
M
L S N Y S R
MS2Scan#: 12669
Energy: 40
Precursor m/z: 1112.13
Precursor charge: 3
Peptide mass: 967.53
Protein ID: P35846
File: WYQ_MusOvary_IGP_F3_20240116
MS2Scan#: 12672
Energy: 27
Glycan mass: 2367.85
Glycan composition: N4H5F3G1
Structure probabilities:
> Core-fucosylation: /
> Bisected GlcNAc: /
> Glycan subtypes: /
> Branch-1: /
> Branches overall: /
N
I T D I E N G T F A N I P R
MS2Scan#: 27159
Energy: 40
Precursor m/z: 1146.53
Precursor charge: 3
Peptide mass: 1788.96
Protein ID: Q99MQ4
File: WYQ_MusOvary_IGP_F2_20240116
MS2Scan#: 27161
Energy: 27
Glycan mass: 1647.61
Glycan composition: N5H3F1
Structure probabilities:
> Core-fucosylation: /
> Bisected GlcNAc: /
> Glycan subtypes: /
> Branch-1: /
> Branches overall: /
Relative Intensity
m/z

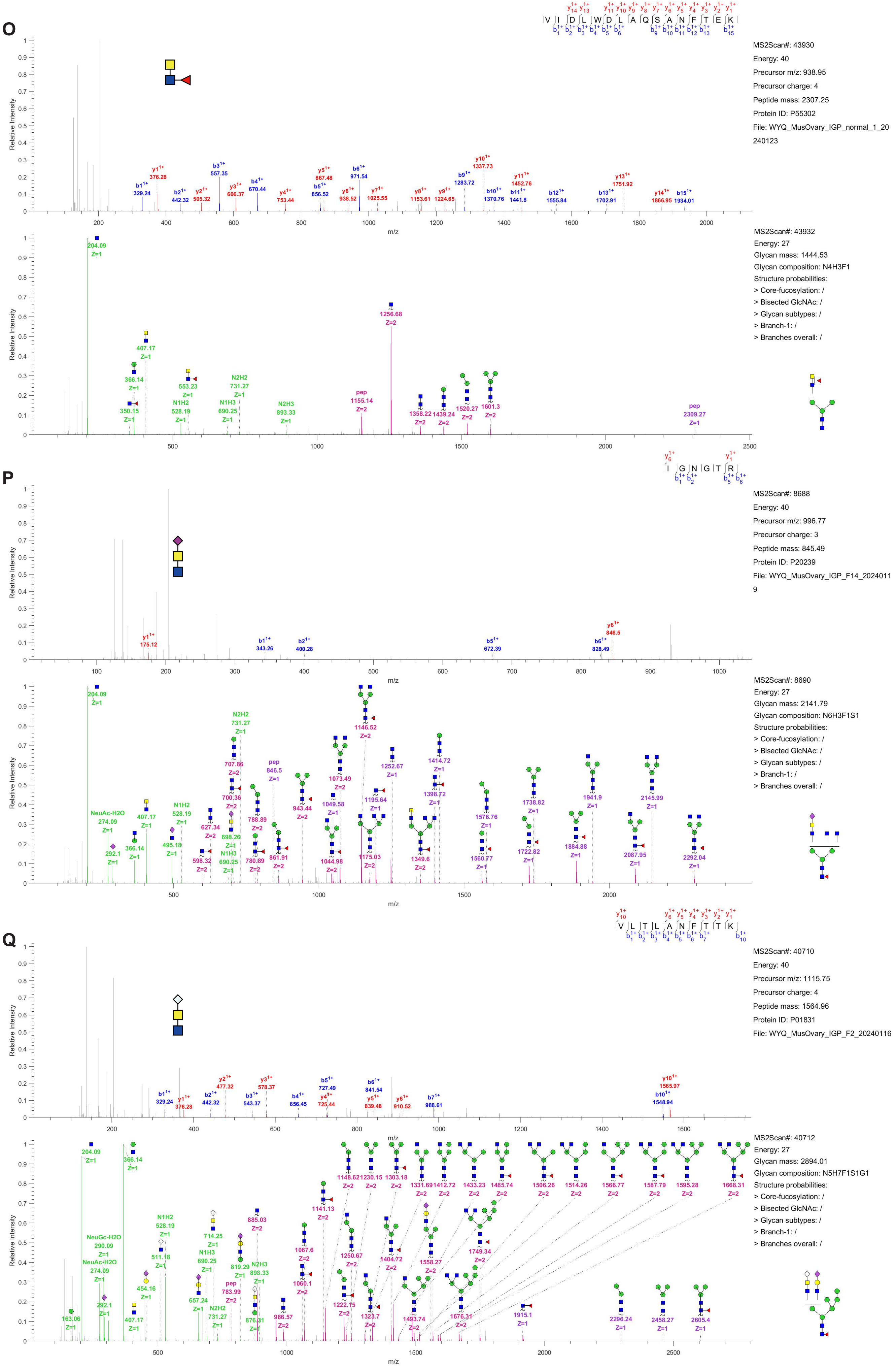

Figure S4 The MS/MS Spectra of three glycan subtypes, four core structures, and seventeen branch structures.
